# Functional correction of *CFTR* mutations in human airway epithelial cells using adenine base editors

**DOI:** 10.1093/nar/gkab788

**Published:** 2021-09-14

**Authors:** Sateesh Krishnamurthy, Soumba Traore, Ashley L Cooney, Christian M Brommel, Katarina Kulhankova, Patrick L Sinn, Gregory A Newby, David R Liu, Paul B McCray

**Affiliations:** Department of Pediatrics, University of Iowa, Iowa City, IA, USA; Department of Pediatrics, University of Iowa, Iowa City, IA, USA; Department of Pediatrics, University of Iowa, Iowa City, IA, USA; Department of Pediatrics, University of Iowa, Iowa City, IA, USA; Molecular Medicine Graduate Program, Pappajohn Biomedical Institute, University of Iowa, Iowa City, IA, USA; Department of Pediatrics, University of Iowa, Iowa City, IA, USA; Department of Pediatrics, University of Iowa, Iowa City, IA, USA; Molecular Medicine Graduate Program, Pappajohn Biomedical Institute, University of Iowa, Iowa City, IA, USA; Merkin Institute of Transformative Technologies in Healthcare, Broad Institute of Harvard and MIT, Cambridge, MA, USA; Department of Chemistry and Chemical Biology, Harvard University, Cambridge, MA, USA; Howard Hughes Medical Institute, Harvard University, Cambridge, MA, USA; Merkin Institute of Transformative Technologies in Healthcare, Broad Institute of Harvard and MIT, Cambridge, MA, USA; Department of Chemistry and Chemical Biology, Harvard University, Cambridge, MA, USA; Howard Hughes Medical Institute, Harvard University, Cambridge, MA, USA; Department of Pediatrics, University of Iowa, Iowa City, IA, USA; Molecular Medicine Graduate Program, Pappajohn Biomedical Institute, University of Iowa, Iowa City, IA, USA

## Abstract

Mutations in the *CFTR* gene that lead to premature stop codons or splicing defects cause cystic fibrosis (CF) and are not amenable to treatment by small-molecule modulators. Here, we investigate the use of adenine base editor (ABE) ribonucleoproteins (RNPs) that convert A•T to G•C base pairs as a therapeutic strategy for three CF-causing mutations. Using ABE RNPs, we corrected in human airway epithelial cells premature stop codon mutations (R553X and W1282X) and a splice-site mutation (3849 + 10 kb C > T). Following ABE delivery, DNA sequencing revealed correction of these pathogenic mutations at efficiencies that reached 38–82% with minimal bystander edits or indels. This range of editing was sufficient to attain functional correction of CFTR-dependent anion channel activity in primary epithelial cells from CF patients and in a CF patient-derived cell line. These results demonstrate the utility of base editor RNPs to repair *CFTR* mutations that are not currently treatable with approved therapeutics.

## INTRODUCTION

Cystic fibrosis (CF) is the most common lethal genetic disease among Caucasians, with over 70 000 persons affected worldwide. Multiple different mutations in the gene encoding the cystic fibrosis transmembrane conductance regulator (*CFTR*) cause this recessive disorder. While advances in small molecule CFTR modulator therapies have improved protein function for many mutations (1–3), ∼10% of people with CF cannot benefit from these strategies (4), including those with nonsense and splicing mutations that prevent complete synthesis of CFTR protein. New therapies for these patients are urgently needed, as clinical trials with drug candidates to treat CF from *CFTR* premature stop codons have not been successful (5,6) and no medications for splicing mutations have been approved yet (7,8).

Recent successes in clinical trials for other genetic diseases (9–16) have renewed interest in gene therapy and gene editing approaches to treat or prevent CF lung disease (17–21), including gene addition approaches using viral and non-viral vectors for DNA and mRNA delivery (20–25). The identification of Clustered Regularly Interspaced Short Palindromic Repeats (CRISPR) nucleases (26–28) increased the pace of discovery for genome editing applications, including CF (29,30). Editing approaches to correct *CFTR* in principle include mutation-specific homology directed repair (HDR) (29,31–34), ablation of splice mutations (35,36), or insertion of a ‘super exon’ by HDR (37–39). However, CRISPR-Cas nuclease-mediated gene editing with HDR is inefficient in most therapeutically relevant cell types, and is highly cell-cycle dependent (40). Most airway epithelia are mitotically quiescent and do not support efficient HDR (41,42). In addition, non-homologous end joining (NHEJ) repair byproducts such as indels typically predominate following double-strand DNA breaks (DSBs) in most cell types, leading to a high ratio of undesired indels to desired repair outcomes following DSB formation from nucleases such as CRISPR-Cas9 (43).

The advent of base editing (44–47) enabled efficient single base pair changes with minimal undesired byproducts, even in non-dividing cells (43). Base editors convert target DNA base pairs in a programmable manner without the introduction of DSBs or reliance on HDR or NHEJ repair processes. By fusing a non-cutting DNA-binding protein such as CRISPR/Cas or a TALE repeat array with natural or laboratory-evolved cytidine or adenine deaminase enzymes, base editors can introduce C•G-to-T•A base changes or A•T-to G•C-base changes (44,45). Base editing has been used to repair disease mutations in human cells including the apolipoprotein E gene variant *APOE4* (44), the β-thalassemia HBB −28 (A > G) (48), and a Marfan syndrome *FBN1* mutation (49). Since a limitation of the CRISPR/Cas system is the availability of an ‘NGG’ or ‘NG’ PAM sequence, subsequent studies expanded the editing opportunities for base editors through the use of non-G PAMs (50,51) and the evolution of novel deaminase domains (50–54).

The ABE class of editors for A•T-to-G•C modifications offer certain advantages. All mammalian stop codons contain at least one adenine residue on both the top and bottom DNA strands (TAG, TAA, TGA), the removal of which would eliminate the stop codon. ABEs typically yield high product purity (typically ≥99% of base-edited products are A•T-to-G•C), and low frequency of indel byproducts (typically ≤5%) (43,45). Additionally, the high genome-wide specificity of ABE7.10, the base editor variant used in this study (45,55), and the low frequency of Cas-independent off-target editing for ABE7.10 (53,56–58) have been previously documented. Remarkably, ABEs have the potential to correct almost half of known human pathogenic point mutations (59). The most recent CFTR mutation database (CFTR2, cftr2.org, 31 July 2020) lists 360 disease causing variants, and of these 67% are point mutations. Of all single nucleotide mutations in CFTR2, 15% could potentially be corrected using CBEs and 46% using ABEs. Recent proof-of-principle studies used ABEs to correct *CFTR* mutations in human intestinal and airway epithelial organoids (60) and a human airway epithelial cell line (61). In these studies, the investigators used electroporation to deliver plasmid DNA or mRNA, respectively.

The use of ribonucleoproteins (RNPs) as gene editing tools offers the potential advantages of rapid onset of effect and transient duration of activity. We hypothesized that the delivery of ABE RNPs to human airway epithelia could produce sufficient on target gene editing to repair *CFTR* mutations and restore CFTR anion channel function. We demonstrate ABE’s ability to precisely recode a single mutant nucleotide residue in *CFTR* and restore electrolyte transport in relevant primary human airway epithelial cells and in CF patient-derived cell lines.

## MATERIALS AND METHODS

Methods, including statements of data availability and any associated accession codes and references, are available in the online version of the paper.

### Adenine base editors and Cas9 nuclease

#### Cas9-ABE

The ABE7.10-SpCas9-NG (45,62) base editor protein employed in this study was purchased as a custom product from Aldevron (Fargo, ND). The optimized NLS sequences used in ABE7.10-SpCas9-NG were reported by Koblan *et al.* and described as ‘BE4max’ and ‘ABEmax’ (63). This base editor includes an optimized NLS architecture (50,63) and a Cas9 variant with expanded PAM targeting capability (64). The recombinant ABEmax-SpCas9-NG protein containing a nuclear localization signal and a His-tag at the N-terminus was expressed and purified according to Aldevron proprietary methodology. Briefly, expression of the recombinant ABE7.10-SpCas9-NG protein was induced in BL21 cells (NEB) using rhamnose, after which the lysis of cells occurred by high-pressure homogenization. The recombinant protein in the soluble lysate obtained after centrifugation was purified using immobilized metal affinity chromatography and cation exchange chromatography. The resulting protein fractions were dialyzed into the final formulation buffer and concentrated via centrifugation. The final formulation buffer was comprised of: 25 mM Tris−HCl pH 7.4, 300 mM NaCl, 0.1 mM EDTA, 1.0 mM DTT, 50% glycerol. The analysis of purity and concentration of the Cas9 protein was by SDS-PAGE and UV−Vis spectrophotometry. The expression plasmid for ABE7.10-SpCas9-NG was deposited with Addgene (plasmid ID #170663).

#### SpCas9-NG nuclease preparation

For the 3849 + 10 kb C > T mutation, the Cas9 nuclease used was from purchased from IDT (Alt-R SpCas9 Nuclease V3, cat #1081058, Coralville, IA). The SpCas9-NG nuclease used for the 3849 + 10 kb C > T mutation was expressed and purified for this study. To prepare the SpCas9-NG nuclease, the coding sequence was cloned into the pET42b plasmid with a 6xHis tag. BL21 Star DE3 chemically competent cells (Invitrogen) were transformed with the plasmid and picked into 2× YT + 25 μg/ml kanamycin for overnight growth at 37°C. The next day, 1L of pre-warmed 2× YT + 25 μg/ml kanamycin was inoculated at OD 0.03 and shaken at 37°C for about 3 h until OD reach 0.8. Culture was cold shocked in an ice-water slurry for 1 h, following which protein expression was induced by the addition of 1 mM IPTG. Culture was shaken at 16°C for 16 h to express protein. Cells were pelleted at 6000 × g for 20 min and stored at −80°C. The next day, cells were resuspended in 30 ml cold lysis buffer (1 M NaCl, 100 mM Tris−HCl pH 7.0, 5 mM TCEP, 20% glycerol, with three tablets of cOmplete, EDTA-free protease inhibitor cocktail (Millipore Sigma, Cat. No. 4693132001). Cells were lysed by sonification at 4°C for a total treatment of 7.5 min, providing time to cool after every 3 s of treatment. Cell lysate was clarified for 20 min using a 20 000 × g centrifugation at 4°C. Supernatant was collected and added to 1.5 ml of Ni-NTA resin slurry (G Bioscience, Cat. No. 786-940, prewashed once with lysis buffer). Protein-bound resin was washed twice with 12 ml of lysis buffer in a gravity column. Protein was eluted in 3 ml of elution buffer (200 mM imidazole, 500 mM NaCl, 100 mM Tris–HCl pH 7.0, 5 mM TCEP, 20% glycerol). Eluted protein was diluted in 40 ml of low-salt buffer (100 mM Tris–HCl, pH 7.0, 5 mM TCEP, 20% glycerol) just before loading into a 50 ml Akta Superloop for ion exchange purification on the Akta Pure25 FPLC. Ion exchange chromatography was conducted on a 5 ml GE Healthcare HiTrap SP HP pre-packed column. After washing the column with 15 ml low salt buffer, the diluted protein was flowed through the column to bind. The column was washed in 15 ml of low salt buffer before being subjected to an increasing gradient to a maximum of 80% high salt buffer (1 M NaCl, 100 mM Tris–HCl, pH 7.0, 5 mM TCEP, 20% glycerol) over the course of 50 ml, at a flow rate of 5 ml per min. 1 ml fractions were collected during this ramp to high salt buffer. Peaks were assessed by SDS-PAGE to identify fractions containing the desired protein, which were pooled and concentrated using an Amicon Ultra 15 ml centrifugal filter (100 kDa cutoff). SDS-PAGE stained with InstantBlue (Expedion, SKU ISB1L) was used to visualize the purity after each step (Supplemental Figure S1). Concentrated protein was quantified using a BCA assay (ThermoFisher, Cat. No. 23227); the final concentration was 86.4 μM.

#### RNP preparation

Guide RNA (gRNA) was prepared by combining the crRNA (IDT) and tracrRNA (IDT, Coralville, IA, catalog #1072532) at equimolar concentrations (100 μM), annealing at 95°C for 5 min and renaturation at room temperature. The RNP was prepared by combining the gRNA and recombinant SpCas9-ABE7.10 protein in PBS, and incubating at room temperature for 15–20 min. The final concentration of gRNA was 26 μM and SpCas9-ABE7.10 was 11 μM in a total volume of 10 ml RNP. The Alt-R SpCas9 Nuclease V3 (IDT, Coralville, IA, catalog #1081058) and SpCas9-NG nuclease variant protein RNPs were prepared similarly. The final concentration of SpCas9 Nuclease V3 and gRNA was 16 μM and 36 μM, respectively in total volume of 15 μl. The final concentration of SpCas9-NG was 12 μM and gRNA was 27 μM in a total volume of 10 μl. The targeted mutations, cell models used, editor proteins employed, and their respective guide RNAs are listed in Table 1.

**Table 1.** Base editors, nucleases and gRNAs employed to modify *CFTR* mutations

Genotype (target mutation/non-target mutation)	Cell line/ primary cells	Gene editing tool	Guide RNAs (gRNA)
**R553X**/ΔF508	CuFi-3	Base editor: ABE7.10max-SpCas9-NG	TTGCTC**A**TTGACCTCCACTC
**R553X**/G85E	Primary	Base editor: ABE7.10max-SpCas9-NG	TTGCTC**A**TTGACCTCCACTC
**W1282X**/CFTR-del2,3	Primary	Base editor: ABE7.10max-SpCas9-NG	CAGTG**A**AGGAAAGCCTTTGG
**3849+10kb C>T**/ΔF508	Primary	Base editor: ABE7.10max-SpCas9-NG	Sp3: GGTG**A**GTAAGACACCCTGAA
**3849+10kb C>T**/ΔF508	Primary	SpCas9 nuclease	Sp2: CTTGATTTCTGGAGACCACA & Sp3: GGTGAGTAAGACACCCTGAA
**3849+10kb C>T**/ΔF508	Primary	SpCas9-NG nuclease	NG: CAGTATTAAAATGGTGAGTA

Target mutations listed in bold. Bold and underlined adenine ‘A’ in gRNA sequence indicates nucleotide target for adenine base editor (ABE) to convert to G.

### Cell models

#### Cell lines

The CuFi-3 cell line is compound heterozygous for ΔF508 and the R553X mutation and was previously described (65). The cells were expanded and maintained in the BEGM media supplemented with 3% fetal calf serum (FCS) media containing penicillin (50 units/ml), streptomycin (50 μg/ml). The cell line was maintained at the air–liquid interface (ALI) on collagen-coated Costar Transwell polycarbonate filters (#CLS3413, 0.3 cm^2^ surface area and 0.4 μm pore size, Corning Inc., Corning, NY). Transepithelial resistance values of the differentiated CuFi-3 CFTR R553X cell cultures were above 300 Ω/cm^2^.

#### Primary cells

Primary human airway epithelial cells with specific *CFTR* mutations were obtained from the University of Iowa's In Vitro Models and Cell Culture Core. Primary CF and non-CF airway epithelia isolated from human donor trachea or bronchi were grown at the air-liquid interface on collagen coated Costar Transwell polycarbonate filters (#CLS3413, 0.3 μm^2^ surface area) as reported previously (66). Cultures were maintained in media supplemented with Ultro-ser G (USG) and the following antibiotics: penicillin (50 units/ml), streptomycin (50 μg/ml). The cultured cells were maintained at 37°C in 5% CO_2._ All primary epithelial cells were well-differentiated (>4 weeks old; resistance > 1000 Ω × cm^2^). Non-CF control primary airway epithelia included one sample from a carrier with one copy of a pathogenic *CFTR* mutation (ΔF508). The study was approved by the Institutional Review Board at the University of Iowa.

### Electroporation of RNPs

Primary cells grown at ALI were trypsinized with TrypLE Express Enzyme (Gibco Laboratories, MD), pelleted at 1000 rpm for 10 min, washed with phosphate buffered saline (PBS) and counted. Approximately 400 000 cells were reconstituted in 100 μl of nucleofection buffer solution (Amaxa Basic Nucleofector Kit for Primary Mammalian Epithelial Cells, Lonza, Switzerland) and mixed with 10 μl of RNP and 1 μl of 100 μM electrophoretic enhancer (IDT, Coralville, IA, catalog #1075915). The cell suspension was electroporated using the Lonza Nucleofector 2b device (catalog #AAB-1001) and a validated human airway epithelial cell program, U-024. After electroporation, pre-warmed Pneumocult Ex-Plus culture media (StemCell Technologies, Vancouver, Canada) was added and the suspension transferred to a single well of a collagen-coated 24-well or 12-well plate. Following 24 h incubation at 37°C in 5% CO_2_, the culture media was changed. At 90% confluency, cells were trypsinized and either reseeded onto collagen-coated polycarbonate Transwell membranes and maintained with the PneumaCult-ALI medium (StemCell Technologies, Vancouver, Canada) or harvested for genomic DNA.

### Analysis of genomic DNA samples

#### Next generation sequencing

Immediately following the electrophysiology testing or one week after incubation post treatment at ALI, genomic DNA was isolated using QuickExtract (catalog #QE09050, Lucigen, Middleton, WI) according to the manufacturer's protocol. The genomic sites of interest were PCR amplified (KAPA DNA polymerase, Roche, Basel, Switzerland)) using primers with homology to the desired regions and the appropriate Illumina forward and reverse adaptors as described (45) (Table 2). Unique Illumina barcoding primer pairs were then added to each sample in a second PCR reaction. Purified PCR products were quantified and then sequenced using a single-end read of 200–250 bases on the Illumina MiSeq instrument using the manufacturer's protocols. Following high throughput sequencing (HTS), the sequencing reads were demultiplexed using MiSeq Reporter (Illumina) and aligned to the appropriate reference genome as previously reported (67). Indel and base substitution frequencies were assessed using the software package CRISPResso2 (67), which counts indels of ≥1 base occurring in a 30-base window around the ABE nicking site. Indels were defined as detectable if there is a significant difference (Student's two-tailed *t*-test, *P* < 0.05) between indel formation in the treated sample and untreated control. Base editing frequencies were further assessed using a previously described MATLAB script (44). For each *CFTR* mutation we determined the target base editing frequencies, bystander edits, and indel frequency for each editor (45),(67). The allelic editing frequency was calculated by the following equation: (% editing-50)/50. The NGS data are accessible through the NCBI sequence read archive (accession: PRJNA745966).

**Table 2.** PCR primer sets for amplifying edited regions

Mutation	Forward primer (adapter sequence is underlined and primer sequence is bold)	Reverse primer (adapter sequence is underlined and primer sequence is bold)
R553X	ACACTCTTTCCCTACACGACGCTCTTCCGATCTNNNN**GGAAGATGTGCCTTTCAAATTCAG**	TGGAGTTCAGACGTGTGCTCTTCCGATCT**ATGTGATTCTTAACCCACTAGCCA**
W1282X	ACACTCTTTCCCTACACGACGCTCTTCCGATCTNNNN **AGAAGTGATCCCATCACTTTTACC**	TGGAGTTCAGACGTGTGCTCTTCCGATCT **TGCAGAGTAATATGAATTTCTTGAGTAC**
3849+10 kb C>T	ACACTCTTTCCCTACACGACGCTCTTCCGATCTNNNN **GGTATAAGCAGCATATTCTCAATAC**	TGGAGTTCAGACGTGTGCTCTTCCGATCT **AGTGTTGAATTTGGTGCTAGCTG**

PCR primer sets for R553X, W1282X, and 3849 + 10 kb C > T were used to amplify the editing window of genomic DNA for Next-generation sequencing.

### Analysis of off target editing

The CRISPOR program (http://crispor.tefor.net) (68) computational tool was used to predict gRNA-dependent off-target editing sites for each mutation (Supplemental Figure S2). The on-target guide RNA associated with each mutation was set as the query sequence with an NGN PAM. From the list generated from CRISPOR, we selected the top 10 predicted off-target sites ranked by cutting frequency determination (CFD) score in descending order. Primers were designed using the IDT primer design software (IDT, Coralville, IA) with the Illumina forward and reverse adaptors as mentioned (see Table 3). Amplified genomic DNA off-target sites were sequenced using next generation sequencing.

**Table 3.** PCR Primer sets for guide RNA-dependent off editing site analysis

	**R553X target gRNA sequence**	**PAM**	Forward primer*	Reverse primer*
OT1	**GA**G**T**TCA**C**TGACCTCCACTC	TGG	TCTCAATAGATGCGTTGCAAATG	CAACCTGTTTATCAAGATCAGCATAC
OT2	**G**TGCTC**G**TT**T**ACCTC**A**ACTC	TGG	ATCCAGCAAAGACAGACCATAG	AGACACATCTGAAGGGCAAAT
OT3	T**G**GCTCA**C**TG**G**C**T**TCCACTC	TGG	CACTGCCAAGAACTTTCTCTACT	TGTGTTCAAAGACATGGAGGAA
OT4	TTG**AC**C**TC**TGACCTCCACTC	AGG	GGTCCAGGCAGAAAGCTAC	GTGAAGCAAGGCTCACAGA
OT5	**AG**GCTC**CC**TGACCTCCACTC	TGG	ACAGACTTCGCCCAAACC	GAGGGAAGGGACACTGGA
OT6	**C**TGCT**T**A**C**TG**C**CCTCCACTC	AGG	AAATAGATCCATGGAGGGCAAG	CCATCTCTTCATGTGGAACTGA
OT7	T**G**GCTC**TC**TGA**A**CTCCACTC	TGG	GGCTGACCCTTCCAAATCTA	GAAGAGAATGACTTCGAGGACT
OT8	**C**TGCTCA**A**TGA**TT**TCCACTC	TGG	CCAGAGTAGGCAAAGAGAAGTG	CTGTCTGAACCCTGGGAAAG
OT9	TT**T**CT**A**ATTGA**A**CTCCA**T**TC	AGG	CCTGCTCAATGTGAGGGTAAT	GTGACCATTGGCAAGAGAGATA
OT10	TTGCTC**CC**TG**G**C**T**TCCACTC	AGG	GGGCAACTACCCACCTAAATAA	CACAGCCAGTCCAAGGTAAA
	**W1282X Target gRNA sequence**	**PAM**	Forward Primer*	Reverse Primer*
OT1	**AT**GTGAAGGAAAGCC**A**TT**A**G	AGG	TGAAGGGAAACTGGCACCTA	CTGATCACTTTGTCTAATCATTCTTTGC
OT2	**A**AGTG**T**AGGAAAGCC**AC**TGG	GGG	ACTTCTGACCTCAGGTGATCT	ACCATGCCCACCAAACTC
OT3	CAGTG**T**AGGAAAGCC**A**TT**AA**	TGG	CAGAAACTACCAAACTTCCTGAAATAG	ATCGTGGGCCATAGTTGTAAAT
OT4	C**G**GT**C**AAGGAAAGCC**A**T**A**GG	AGG	CACTTGTTATAGGTCTGTTGAGATTG	TGGCCTCTACTGTGTTTCTTAC
OT5	CAGTGAA**T**G**T**AAGCCT**C**TG**A**	TGG	CAAGCCACCTCTGGGTAAA	GTTCAGCAGTGCAAGCAATAG
OT6	**A**AGTGAAG**C**AA**G**GCC**A**TTGG	TGG	ACCTGGTGAGCCTCATTTC	CTTATCTTCTCCTCTTGGTAGATAAGTA
OT7	CAGTGAA**AT**AAAGCC**A**TT**A**G	TGG	CTGCATGTCATCTCTTCCTCTT	CTAAAGCATCATTTGCCAGGATAA
OT8	CAGT**AT**A**A**GAAAGCCT**A**TGG	AGG	CCCAGACTAGATCCTATGCTTTG	ACATCCCGTCTTGTTGTGAA
OT9	**G**AGTG**TG**GGAAAGCCTTT**A**G	TGG	TCGAAAGGTCTTCCCACATTC	GCTTTCATCCGCAAGTCAAAC
OT10	CAGTG**T**AGGAAAG**T**CTTT**TA**	GGG	ACTCTAATTCACTGGGCCATTT	GTACTCCCATCTCACCTCTTTATTC
	**3849 Target gRNA sequence**	**PAM**	Forward Primer*	Reverse Primer*
OT1	**A**GTGA**A**TAA**AG**CACCCTGAA	AGG	CCATTTAGAAACAGCAGCACAA	GGTCCTATAGCTCATCCACAATC
OT2	GGTGA**A**T**GT**GACACC**A**TGAA	TGG	TTGGTCAGATGTCAGGAGAAAC	CACTCTAGGAGGAAGGGACTATTA
OT3	**A**GTGAG**G**AA**A**ACACCCT**A**AA	AGG	AGTGTACTCTTCATTGAATCCTAGT	GAGGACACTGCTCCTAATTCC
OT4	**T**GTG**G**GTA**T**GA**A**ACCCTGAA	AGG	GGGCTGGGCTAAGCAATAAT	CAGGTGAAAGTGTCAGGTAAGG
OT5	**AA**TGAGTAAGACA**T**CC**A**GAA	GGG	CATTTGTGAATTCTCCCGTCATT	GAGACCACCTCCTTCCTTTG
OT6	**A**G**G**GA**A**TAAG**G**CACCCTGAA	GGG	CATCTGTGGACATCCTGTCTTT	TGTAACCCATGTGCCAAGTTA
OT7	G**AA**GA**A**T**T**AGACACCCTGAA	CGG	AACACCTCTACACCCGTAAAC	TCGTACTTTGAATGTCTGGTAGAA
OT8	GGTG**G**G**C**AAGA**T**ACCCTGA**G**	GGG	GTCCTGCAAAGTGCTACCTAA	ATCGCATTGCACCTCAATTTC
OT9	**A**GTGAGT**C**A**TG**CACCCTGAA	GGG	ATGTGAGCTCTGTGCTTGG	AGATGCCAGTCACCCTTTG
OT10	GG**GA**AG**C**AAG**G**CACCCTGAA	TGG	CAAAGTTATCTTAGTTTCTTGGTGGTC	GTAAAGTGACTGGAGATAGAAGCTC

* Adapters were added to the primers in the following format:

ACACTCTTTCCCTACACGACGCTCTTCCGATCTNNNN-Forward Primer Sequence

TGGAGTTCAGACGTGTGCTCTTCCGATCT-Reverse Primer Sequence

See Supplemental Figure S2.

Note: The bold lettering in the gRNA sequences denotes the differences between the on-target guide RNA sequences and the off-target guide RNA sequences.

### Electrophysiology

Well-differentiated airway epithelial cultures were mounted in the Ussing chambers and assessed for a change in short circuit current in response to stimuli as previously described(69). Epithelial cultures were pre-stimulated overnight with cAMP agonists forskolin (Cayman Chemical, Ann Arbor, MI) (10 μM) and 3-isobutyl-1-methylxanthine (IBMX) (Sigma Aldrich, St. Louis, MO) (100 μM) (F&I). Next, cultures were mounted in the Ussing chambers, bathed in symmetrical Ringers solution (135 mM NaCl, 5 mM HEPES, 0.6 mM KH_2_PO_4_, 2.4 mM K_2_HPO_4_, 1.2 mM MgCl_2_, 1.2 mM CaCl_2_, 5 mM dextrose), transepithelial voltage (V_t_) was maintained at 0, and baseline currents were established. CFTR current was measured using the following protocol: amiloride (both Sigma Aldrich, St. Louis, MO) (100 μM), 4,4’-dilsothiocyano-2,2’-stilbenedifulonic acid (DIDS) (Sigma Aldrich) (100 μM), F&I, followed by the CFTR channel inhibitor GlyH-101 (GlyH).

### Statistical analysis

Student's two-tailed *t*-test, one-way analysis of variance (ANOVA) with Tukey's multiple comparison test, or two-way ANOVA with Dunnett's multiple comparison test were used to analyze differences in mean values between groups. Results are expressed as mean ± SE. *P* values ≤0.05 were considered significant.

## RESULTS

### Adenine base editors modify CFTR mutations with high fidelity and minimal bystander events

**Figure 1.**
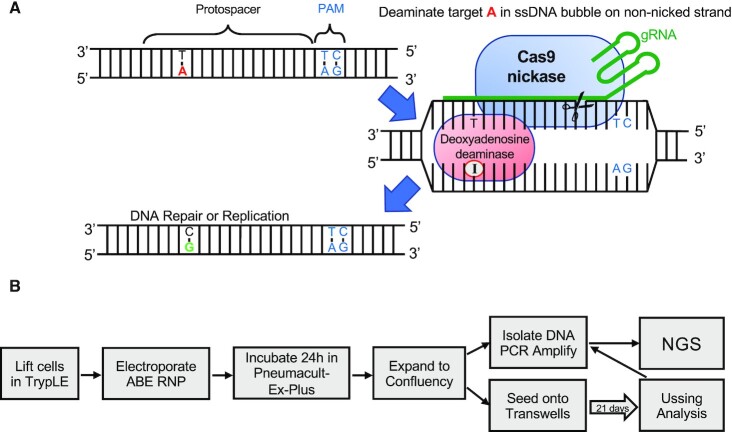
Experimental design. (**A**) Schematic of adenine base editor (ABE)-mediated A•T to G•C base editing. ABEs contain a deoxyadenosine deaminase and a catalytically inactive SpCas9 with nickase activity. The ABE binds target DNA in a guide RNA (gRNA)-programmed manner, exposing a small bubble of single-stranded DNA. The deoxyadenosine deaminase catalyzes conversion of adenine to inosine within this bubble. Following DNA repair or replication, the original A•T base pair is replaced with a G•C base pair at the target site. (**B**) Diagram of workflow for ABE delivery to airway epithelia and methods of downstream analysis.

We used a catalytically inactive *S. pyogenes (Sp)* Cas9(26) CRISPR variant ‘SpCas9-NG’(64) with nickase activity to direct the adenosine deaminase enzyme to specific genomic target sequences (see schematic, Figure 1A). The SpCas9-ABE7.10 protein requires an ‘NG’ protospacer adjacent motif (PAM) located directly downstream and on the opposite strand of the genomic DNA target sequence (45,50). Depending on the available PAM sites, ABE proteins may yield variable editing efficiencies and bystander edits (unwanted A to G alterations within the editing window) for targeted and non-targeted A•T-to-G•C conversions. The targeted mutations, cell models used, editor proteins employed, and their respective guide RNAs are listed in Table 1. We evaluated the editing efficiency for the following disease-causing mutations: R553X (c.1657C > T), W1282X (c.3846G > A), and 3849 + 10kb C > T (c.3718–2477C > T).

**Figure 2.**
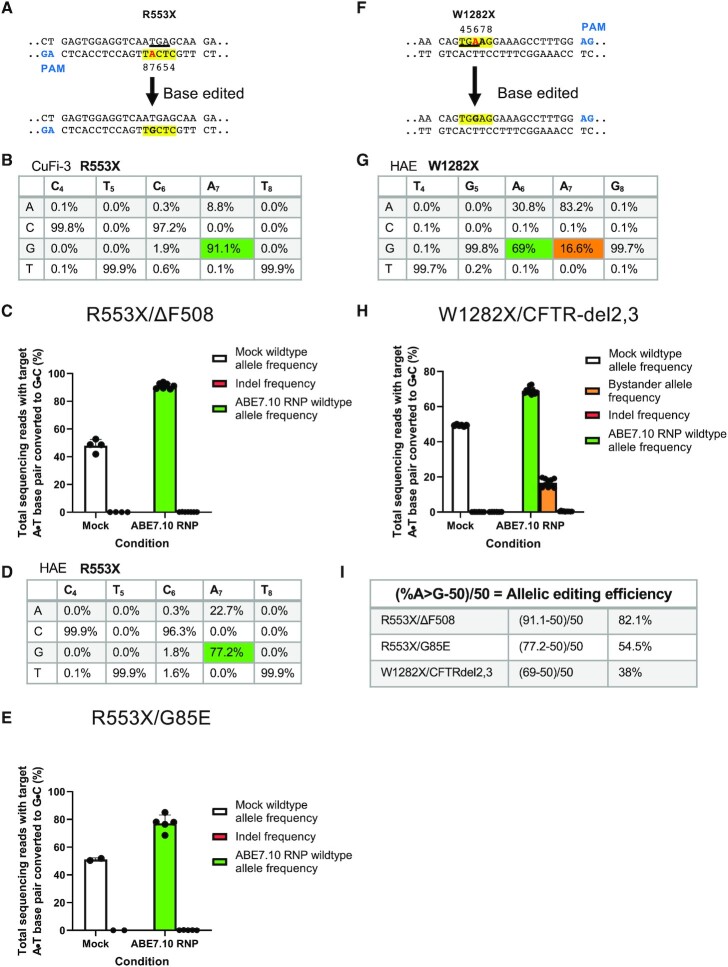
Frequency of desired product and allelic editing efficiencies for *CFTR* nonsense mutations. One week following Cas9-ABE RNP delivery, DNA was analyzed by next generation sequencing. (**A**, **F**) Top panels show the target DNA strands and PAM sites (blue text). Yellow highlight denotes the predicted 4–8 nt ABE editing window (numbered). Mutations highlighted in red text. (**F**) Bold **A** in position 7 indicates the critical bystander mutation Lower panels display the nucleotide sequences within the editing window following A•T to G•C editing. (**B**, **D**, **G**) NGS results: nucleotide frequency within the target editing window after base editing. The A•T to G•C nucleotide frequency is noted by the boxed numbers. The frequency of the desired product at target site is highlighted in green. ABE A•T to G•C editing of the *CFTR* R553X mutation in the CuFi-3 cell line (B), R553X HAE (D) and W1282X (G). For W1282X, a bystander edit immediately adjacent to the target mutation was observed at a frequency of 16.6% (orange box in (G). (**C**, **E**, **H**) Total sequencing reads, bystander editing, and indel frequencies for each genotype quantifies the A•T to G•C conversion for (C) CuFi-3 R553X (*n* = 7), (E) R553X primary human airway epithelia (HAE) (*n* = 5), and (H) W1282X (*n* = 9). (**I**) The allelic editing efficiency for each mutation calculated by (% editing-50)/50. Untreated control cells (*n* = 6) had the expected 100% mutant allele frequency. Each data point represents one airway culture.

To deliver SpCas9-ABE7.10 RNPs to CF airway epithelia we used electroporation. The experimental workflow is described in Figure 1B. We first investigated this approach in the CuFi-3 cell line that is compound heterozygous for R553X/ΔF508. The R553X has a single A nucleotide within the editing window. One week after electroporation we isolated genomic DNA, PCR-amplified the target region, and used next-generation sequencing (NGS) to assess editing (Figure 2A). Following ABE editing, we observed a 91.1% frequency of the desired product (G nucleotide at A_7_), corresponding to an allelic editing efficiency of 82.1% when accounting for the 50% of reads that started with a G in this position, with no bystander edits and little evidence of indels (0.11%) (Figure 2B, C, I). Because these cells are compound heterozygotes at the targeted A→G nucleotide, which has a frequency of 50% G in unedited control cells, the total frequency of the desired outcome at this position and the editing efficiency are not identical. We calculated the allelic editing efficiency by subtracting the 50% starting frequency of G from the observed frequency after editing, then divided by the maximum possible value of 50%. For example, an observed frequency of 80% of the desired G nucleotide after editing means that (80–50)/50 = 60% allelic editing efficiency was achieved, and thus we can infer that 60% of cells had been edited to contain one wild-type allele. Targeting the same mutation (R553X), an identical approach was used to deliver the SpCas9-ABE7.10 RNPs to primary CF airway epithelia bearing the R553X/G85E mutations. Here, we achieved a 77.2% frequency of the desired product (G nucleotide at A_7_) for an overall allelic editing efficiency (A•T to G•C) of 54.5%, no bystander edits, and an indel frequency of ≤1% (Figure 2D, E, I). These experiments demonstrate that ABE RNPs corrected the R553X mutation in both an immortalized cell line and primary airway epithelia.

We also delivered SpCas9-ABE7.10 RNPs to primary human airway epithelial cells compound heterozygous for the W1282X and CFTR-del2,3 mutations. The W1282X mutation presents challenges for base editing as there are two A nucleotides within the editing window, the target nucleotide at position A_6_ and a bystander nucleotide at position A_7_ where editing would lead to a undesired nonsynonymous change, R1283G (Figure 2F (bold A_7_),G). After electroporating the base editor, then sequencing genomic DNA by NGS, we observed that the frequency of the desired product (G nucleotide at A_6_) increased from 50% in unedited, heterozygous controls to 69%, corresponding to an allelic editing efficiency of 38% of cells (Figure 2I). The frequency of a G nucleotide at bystander position A_7_ increased from 0% in unedited controls to 16.6% following treatment (Figure 2G,H). Of note, the A > G changes occurring at position A_7_ only arose in conjunction with the A > G changes at position A_6_. This suggests the bystander edit cannot occur as a standalone edit.

**Figure 3.**
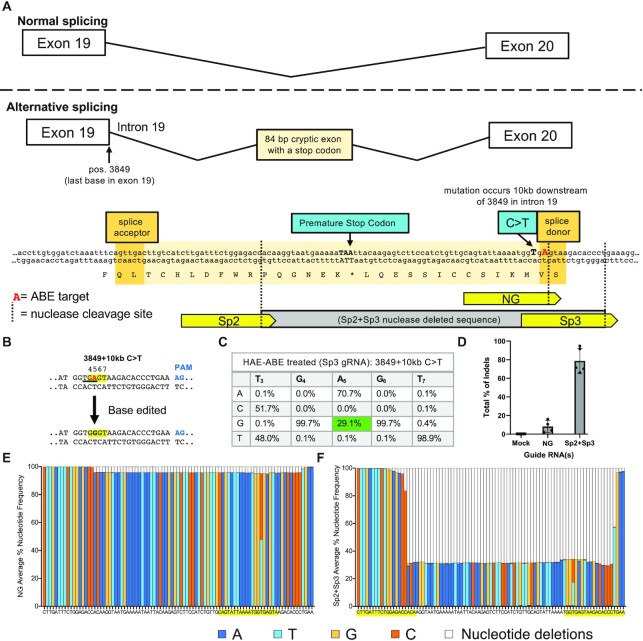
Modifying the 3849 + 10 kb C > T mutation. (**A**) Schematic of 3849 + 10 kb C > T mutation site in *CFTR* intron 19. Top panel: normal exon splicing results in generation of exons 19 and 20. Bottom panel: Alternative splicing schematic. The indicated C > T mutation in the intron 10 kb downstream from the end of exon 19 (pos. 3849) creates a partially active splice donor site within intron 19 that can result in the insertion of a new 84 bp cryptic exon containing an in-frame stop codon between exons 19 and 20. Guide RNAs are shown in yellow and labeled Sp2, Sp3 and NG (see Table 1). For single cleavage with SpCas9-NG or dual cleavage with SpCas9 + Sp2 and Sp3 gRNAs, the gray bar indicates the expected deleted sequence. Vertical dotted lines indicate predicted DNA cleavage sites for nuclease-treated samples. Red colored ‘A’ denotes target for ABE treated cells. (**B**) Target DNA strands and PAM sites (blue text). Yellow highlight denotes the predicted 4–8 nt ABE editing window (numbered). Targeted A•T to G•C nucleotide (red text) change after base editing disrupts splice donor. Lower panel displays the nucleotide sequences within the editing window. (**C**) Frequency of desired product following ABE delivery. The on-target frequency of the desired product at the target site is highlighted in green, bystander allele frequency in orange (*n* = 3). (**D**) Percentage of indels generated using a Cas9 nuclease approach. (**E**) Sequencing alignment of NG sgRNA following Cas9 nuclease activity (*n* = 4). F) Sp2 and Sp3 gRNAs delivered with Cas9 resulted in a ∼60 nt deletion. Sequencing alignments are shown (*n* = 5).

To disrupt the novel splice site created by the 3849 + 10 kb C > T mutation, we compared the use of ABE RNP and CRISPR/Cas nuclease approaches (see Table 1). A schematic of this mutation and the gRNA targets is shown in Figure 3A. We note that for this intronic mutation, the editing strategies are not allele specific. While the C > T mutation is not amenable to ABE modification, a nearby A nucleotide is part of the consensus splice donor site motif (G**T**RAGT) and a candidate for deamination (highlighted in red in Figure 3A). Following ABE delivery to edit the splice donor sequence (Sp3 in Figure 3A), we observed a 29.1% frequency of the desired product (G nucleotide at A_5_) and bystander editing frequency of 1.8% (Figure 3B, C). The editing efficiency for nuclease-mediated activity with a single cleavage approach using the NG gRNA was 8.2% (Figure 3D, E). The SpCas9 nuclease strategies for disrupting the aberrant splice site were most successful when a dual cleavage approach was employed, deleting ∼60 nt and excising the C > T mutation (Sp2 + Sp3, schematically shown by gray bar in Figure 3A). We observed an editing efficiency of 79% for the mutant alleles (Figure 3D, F). Indels were observed on both alleles. Overall, we observed editing efficiencies of 38–82.1% among all three mutations targeted using both ABE and nuclease-mediated approaches. We used the CRISPOR computational tool (68) to identify sites for predicted gRNA-dependent off-target base editing (Supplemental Figure S2A). Following sequencing these genomic loci, we found no evidence of off target editing for all four cell types treated with SpCas9-ABE7.10 RNPs (Supplemental Figure S2B–E).

### Gene editing restores CFTR anion channel function

**Figure 4.**
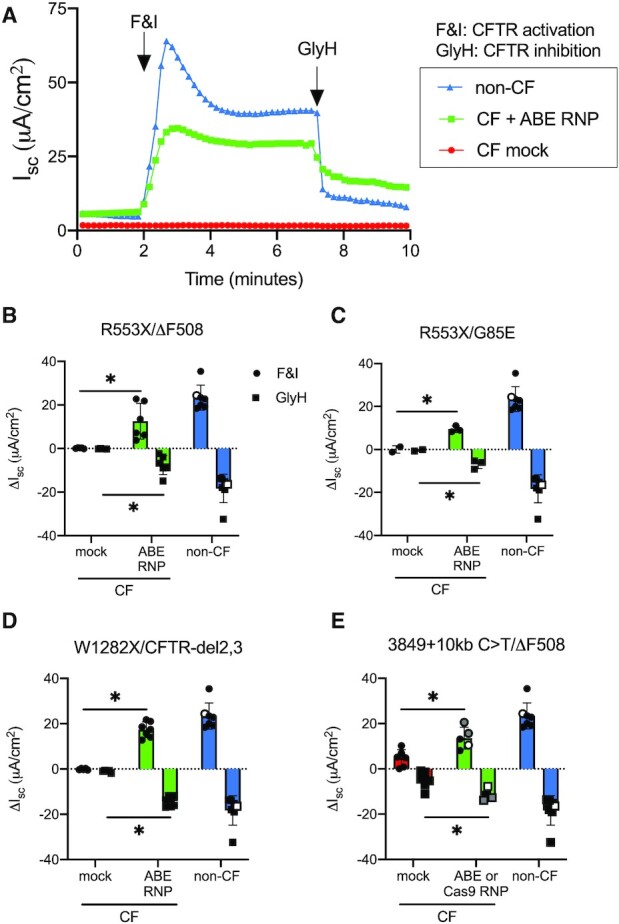
ABE RNP delivery modifies three *CFTR* mutations and restores CFTR-dependent anion transport defect. Gene editing reagents targeting individual mutations were delivered as described in Materials and Methods. At three or more weeks after electroporation, CFTR-dependent anion channel activity (Δ*I*_sc_= change in short circuit current) in response to F&I = forskolin and IBMX (activates CFTR) and GlyH = GlyH-101 (inhibits CFTR) was measured in Ussing chambers. (**A**) Representative short circuit current tracing of ABE treated CuFi-3 (R553X/ΔF508) cells comparing mock and ABE RNP electroporated cells to non-CF control epithelia. Note: data from non-CF donors (*n* = 7) are shared in panels (B–E). Results from one Het control epithelium (ΔF508/wild type) are indicated by white symbols. (**B**) CuFi-3 cells with the R553X/ΔF508 mutation were assessed for anion channel activity (*n* = 6). (**C**) Anion channel current measured on primary human airway epithelial (HAE) cells with the R553X/G85E mutation (*n* = 3). (**D**) W1282X/CFTR-del2,3 (*n* = 7) and (**E**) 3849 + 10 kb C > T/ΔF508 (*n* = 5). For panel E: gray symbols: SpCas9 + Sp2 and Sp3 gRNAs; black symbols: ABE + Sp3 gRNA; white symbols: SpCas9-NG gRNA (see Table 1). Each data point represents one culture, *P< 0.05.

To evaluate the functional impact of editing *CFTR* mutations, we performed studies of CFTR-dependent anion channel activity in Ussing chambers. We measured CFTR-dependent short-circuit current >21 days after editor delivery. We first studied the impact of ABE on the R553X mutation in CuFi-3 cells (R553X/ΔF508) and primary airway epithelia compound heterozygous for the mutation (R553X/G85E). Figure 4A shows a representative short-circuit tracing for ABE treated CuFi-3 cells (R553X/ΔF508) in comparison with mock electroporated R553X/ΔF508 cells, and non-CF epithelia. Figure 4B and C summarizes the results from edited R553X/ΔF508 and R553X/G85E epithelia, respectively. Following the sequential addition of amiloride to inhibit epithelial sodium channels (ENaC) and DIDS to inhibit non-CFTR Cl^–^ channels, we applied forskolin and IBMX (F&I, cAMP agonists) to activate CFTR-dependent Cl^–^ secretion (measured as change in short circuit current, Δ*I*_sc_). F&I increased intracellular cAMP levels leading to the PKA-mediated phosphorylation and activation of CFTR channels. Activation of CFTR was subsequently confirmed by the addition of the CFTR channel inhibitor GlyH-101 (GlyH). In both cell types, CFTR-dependent Cl^–^ transport increased significantly following ABE editing.

Ussing chamber studies of edited W1282X/CFTR-del2,3 epithelia also demonstrated restoration of CFTR-dependent Cl^–^ secretion that was GlyH sensitive (Figure 4D). We note that the CFTR-del2,3 mutation results in the complete loss of exons 2 and 3 from the transcript causing a functional null for that allele (70). Thus, the observed restoration of CFTR function observed following ABE editing solely arises from the modified W1282X allele.

For cells with the 3849 + 10 C > T mutation we observed phenotypic correction using both ABE and CRISPR/Cas9 approaches (Figure 4E; gray symbols: SpCas9 + Sp2 and Sp3 gRNAs (see Table 1 and Figure 3A); black symbols: ABE + Sp3 gRNA; white symbols: SpCas9-NG gRNA). These cells had the ΔF508 mutation on the other allele. Because of variable aberrant splicing, the 3849 + 10 kb C > T mutation yields some normally spliced *CFTR* mRNA and variable baseline Cl^–^ secretion. We observed significant increases in Cl^–^ transport following gene editing, with the greatest recovery of activity arising from the cells treated with SpCas9 + Sp2 and Sp3 gRNAs. Importantly, these data indicate that electroporated SpCas9-ABE7.10 RNPs or CRISPR nucleases can edit this genomic locus efficiently enough to confer physiologic correction.

**Figure 5.**
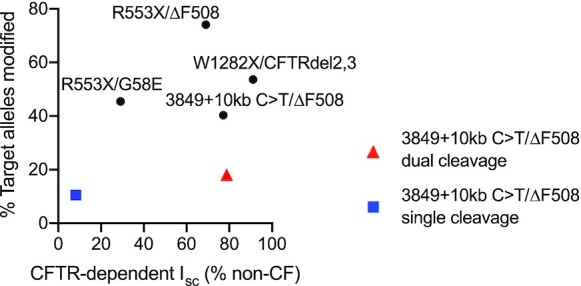
Correlation between the frequency of the desired editing product and restoration of CFTR-dependent Cl^–^ transport. The Y-axis represents the percentage of alleles with the pathogenic mutation eliminated for the ABE or Cas9 nuclease methods applied. Results from ABE editing as reported in Figures 2 and 3 are represented by black circles. Results for the nuclease treated 3849 + 10 kb C > T epithelial cells using single cleavage with SpCas9-NG are represented by a blue square and dual cleavage with SpCas9 + Sp2 and Sp3 gRNAs denoted by a red triangle. R553X/ΔF508 indicates the CuFi-3 cell line. The remainder of results are from primary cultures of human airway epithelia with R553X, W1282X, or 3849 + 10 kb C > T mutations. The X-axis represents the measured CFTR-dependent *I*_sc_ (% non-CF) from Figure 4.

To evaluate the relationship between gene editing and restoration of CFTR-dependent anion transport we plotted the percentage of the A > G editing efficiency versus the non-CF Δ*I*_sc_ (F&I) from edited cells for each mutation studied (Figure 5). Following ABE editing, the % non-CF F&I-dependent Cl^–^ current increased for all mutations edited. The overall trend is one of higher editing efficiencies correlating with greater restoration of CFTR function, but the results were variable based on the mutation type. Importantly, for each mutation evaluated, we attained functional correction within a range believed to be therapeutically relevant. While we observed the highest allelic editing efficiencies with the R553X target, 82% in CuFi-3cells and 54% for primary HAE, the level of CFTR-dependent anion channel correction attained was less than that seen for W1282X, where the allelic editing efficiency was 38%. In the case of 3849 + 10 kb C > T nuclease treated conditions, the dual cleavage method that led to more efficient deletion of the aberrant splice site conferred greater anion channel activity than the single cleavage strategy (Figure 5, red and blue symbols, respectively).

To address the variable correlations between the editing efficiencies and the resultant CFTR-dependent Cl^–^ current, additional studies were performed using CuFi-3 cells (R553X/ΔF508). Following bulk electroporation of cells with SpCas9-ABE7.10 RNPs, we performed single cell cloning and identified cells with 100% allele specific editing of the R553X mutation. As shown in Supplemental Figure S3, compared to bulk electroporated cells, the epithelium derived from fully corrected cells demonstrated increased CFTR-dependent anion transport within the range of the non-CF control cells. These results demonstrate that fully correcting one mutant allele in epithelial cells bearing two deleterious CFTR mutations is sufficient to restore Cl^–^ secretion to non-CF levels.

## DISCUSSION

Adenine base editors have the potential to correct nearly half of all the known single nucleotide *CFTR* mutations, including nonsense and splice-site mutations not amenable to potentiator or corrector treatments. Here, we focused on three mutations unresponsive to current small molecule therapies using human airway cell lines and primary cultures of airway epithelia from people with CF. Further, we selected mutations based on the availability of PAM sequences and their proximity to adenine nucleotides and the availability of primary cells with these mutations. The base editors used here are derived from SpCas9-ABE7.10 (45) and include modifications that improve their editing efficiency via an optimized NLS, codon identity, and improved deaminase domains (63). The engineered variant SpCas9-NG expands the targetable PAM sequences by using the motif ‘NG’ as opposed to the traditional ‘NGG’. We selected a Cas9-ABE that targets a PAM to position the adenine deaminase within an optimal editing window (62). In addition, the Cas9-ABEs include a nickase that directs DNA repair to the non-deaminated strand, a feature believed to improve the editing efficiency up to 90% (44). We quantified A•T to G•C editing and demonstrated that ABEs can functionally correct three clinically important mutations.

Base editing approaches to modifying *CFTR* mutations have some specific utility for respiratory epithelia. While CRISPR/Cas nuclease gene editing with homology directed repair templates is inefficient in quiescent cells, a base editing approach active in airway epithelia would increase the opportunity for gene repair (40). In the inner ear of mice, Yeh *et al.* observed efficient base editing following delivery of Cas9-CBE RNPs to the quiescent post-mitotic support and hair cells (43). Levy *et al.* reported editing efficiencies of 60% in adult mouse cerebral cortex using ABE delivered with AAV vectors (71). These are notable results for lung disease applications as most airway epithelia, including progenitor cell types, are also quiescent (41,42). There is some evidence that the mitotic indices of epithelia increase in the inflammatory state associated with chronic CF lung disease (42). It will be important to understand the efficiency of base editing and cell targeting in airway epithelia in both dividing and non-dividing states.

Two recent proof-of-principle studies used ABEs to correct *CFTR* nonsense mutations. In these studies, the investigators used electroporation to deliver plasmid DNA (60) or mRNA (61), respectively. Geurts *et al.* delivered an ABE expression cassette to human intestinal and airway epithelial organoids bearing the *CFTR* W1282X, R785X, R553X (intestinal), R1162X (nasal airway) mutations using plasmid electroporation (60). They clonally selected edited cells and demonstrated on target editing and functional restoration of CFTR activity using a forskolin-induced organoid swelling assay. Jiang and colleagues delivered ABE mRNA via electroporation to a human airway epithelial cell line with the W1282X CFTR mutation (61). Following clonal selection they identified cells with error free restoration of the tryptophan codon and CFTR channel function. Some edited clones had a bystander edit, in this case Q1281R. In contrast to those studies, we used electroporation for ABE RNP delivery and performed our analysis on the bulk population of edited epithelia, including primary cells. Delivery of ABEs in the form of RNPs may offer advantages due to their rapid onset of effect and transient activity.

A key question for genome editing approaches is to determine how many epithelial cells must be corrected to modify CF phenotypes and which cell types should be targeted. For all compound heterozygous *CFTR* mutations we studied, partial correction of one allele was sufficient to recover CFTR activity. Thus, less than complete correction of one mutant allele may be therapeutically relevant. For cells bearing the 3849 + 10 kb C > T mutation, baseline low level Cl^–^ secretory currents likely reflect variable levels of normally spliced mRNA (72). This is consistent with the clinical phenotype which is characterized by pancreatic sufficiency, male fertility, and later onset of lung disease (73,74). In this case, the level of gene correction required to improve anion transport would likely be less than that needed for a nonsense mutation. Previous studies in which CF and non-CF cells were mixed in various proportions suggest that expression of CFTR in 10–50% of cells is sufficient to obtain near wild-type levels of Cl^–^ secretion (75). Furthermore, epithelial cells with a deleterious mutation on one allele and a normal second allele have CFTR-dependent Cl^–^ secretory currents that are similar to non-CF cells (75). In addition, people with mutations associated with as little as 10% residual CFTR function may have mild disease phenotypes, including little or no lung disease (72).

There are several possible explanations for the observed variability in the correlation between base editing efficiency and correction of CFTR function. Notably, there is increasing evidence that CFTR function in airway epithelia is highly cell type dependent. Not all cell types participate equally in Cl^–^ secretion. Recent scRNA-seq studies have elucidated a diversity of cell types in the large airway surface epithelium (basal, secretory, goblet, club, ciliated, ionocyte, neuroendocrine, hillock, etc.) (76,77). The *CFTR* transcript abundance varies quite widely among individual cell types. Therefore, depending on which cell types are appropriately edited, bulk edited cell populations might be expected to have variable restoration of CFTR-dependent Cl^–^ transport. The cell types of the surface epithelium express different complements of ion channels and transporters that may enhance or counter CFTR-dependent Cl^–^ secretion. A recent study using scRNA-seq, scRNA *in situ* hybridization, and single cell RT PCR demonstrated that secretory cells are the dominant airway surface cell type for CFTR expression and function (78). While ionocytes express abundant *CFTR* transcripts, they are a rare cell type. In contrast, ciliated cells exhibited low and infrequent *CFTR* expression (78). Additionally, ∼30% of the cells in cultured surface airway epithelia are basal cells. Basal cells do not have a luminal membrane expressing CFTR and while they may express *CFTR* transcripts (78), their role in Cl^–^ transport is currently unknown. We also note that the percentage of ciliated and secretory cell types in cultured primary airway epithelia vary considerably and may be donor and disease state dependent.

The only undesired outcome observed at appreciable levels using these editing strategies was at the bystander A_7_ adjacent to the target nucleotide when correcting the W1282X mutation. The deamination of the A_7_ nucleotide introduces the R1283G mutation, a rare but documented mutation associated with a CF phenotype in one subject (www.genet.sickkids.on.ca/cftr). This bystander edit could also occur on the other allele, in this case CFTR-del 2/3. Adenine base editing could potentially result in unintended sgRNA-dependent or sgRNA-independent off target deamination. To address this possibility we used a computational strategy to identify sites of guide RNA-dependent deamination for each mutation studied. We were reassured to find no evidence of off target editing (Supplementary Figure S2). Geurts *et al.* used CIRCLE-seq to assess for off target editing and concluded that adenine base editors caused no detectable off-target effects during repair (60). We note that base editor deaminase variants have been evolved or engineered that have strong sequence context preferences (54,79). Further development of more specific ABE deaminases may improve the specificity of editing outcomes at this site.

The delivery of ABE in the form of RNP protein may offer advantages over various DNA and RNA expression vectors. Because the duration of treatment is short and ends immediately when the protein is degraded, immunogenicity and any off-target editing should be minimized. We delivered the Cas9 nucleases and ABE RNPs by electroporation for proof of principle, however other delivery approaches have yet to be investigated in our model. Amphiphilic peptides are promising delivery tools and rapidly enter airway epithelia (80,81). This method of delivery has been validated in primary cultures of human airway epithelia and in the airways of mice (80). This approach allows for protein delivery and rapid onset of editing. AAV vectors encoding base editors have been successfully used to edit mouse models, circumventing the packaging limit by splitting editor genes in two parts that are joined by a split intein (71). Lipid nanoparticles (LNPs) have also been used to deliver these editing tools (43) and should be assessed for the ability to correct these mutations in animal models.

We acknowledge that there are alternative strategies for ameliorating the effects of CFTR nonsense and splicing mutations. Gene therapy approaches based on delivery of an episomal or integrating CFTR expression cassette are mutation-agnostic strategies under investigation (17,20). For premature termination codons, small molecules to achieve PTC readthrough (82,83) and inhibitors of nonsense mediated decay are alternative approaches. For splicing mutations, antisense oligonucleotides offer a promising strategy for restoration of function (84). While these pharmacologic approaches offer promise, they likely require lifelong continuous therapy.

In contrast, if progenitor cell populations could be efficiently corrected using ABEs, the effect would be long-lived. The CF defects mainly involve the airways and therefore the focus of most gene therapy strategies is delivery of cargoes to epithelial cells of the proximal large airways (pseudostratified columnar epithelium; cartilage and submucosal gland containing) and the small airways (simple columnar epithelium; devoid of cartilage). The progenitor cell types vary regionally in the conducting airways (85). Within these airway regions progenitor cell types include basal cells (K5^+^, p63^+^, Muc5AC^–^) in the proximal cartilage containing tracheobronchial epithelium (86,87), and club cells, basal cells, and α6β4^+^ cell populations in the small airways (88,89). Studies from animal models provide some insights regarding turnover of surface epithelial cell types. In mice the ciliated cells of the large and small airways are surprisingly long-lived (half-life of 6 months in the trachea and 17 months in bronchioles (90)). Detailed information concerning airway cell turnover in humans is not available.

Base editing offers a means to repair rare *CFTR* mutations that cannot be restored by current therapeutic approaches. Several questions must be addressed to advance base editing as a therapy for CF as this field evolves. Which base editor platform offers the best opportunity to achieve the desired nucleotide change? What is the most efficient delivery method to target cells of interest? What cell types can be modified with editors? Will the desired modifications translate *in vivo* and modify phenotypes? In conclusion, we identified candidate base editor RNPs for *CFTR* mutation repair and demonstrate a broadly applicable gene correction strategy for CF and other genetic diseases.

## DATA AVAILABILITY

All relevant data supporting the key findings of this study are available within the article and its Supplementary Material files or from the corresponding authors upon reasonable request. The source data for all figures are provided with the paper. The NGS data are accessible through the NCBI sequence read archive (Accession: PRJNA745966).

## Supplementary Material

gkab788_Supplemental_File

## References

[1] Davies J.C. , Moskowitz S.M., Brown C., Horsley A., Mall M.A., McKone E.F., Plant B.J., Prais D., Ramsey B.W., Taylor-Cousar J.L. et al. VX-659-tezacaftor-ivacaftor in patients with cystic fibrosis and one or two Phe508del alleles. N. Engl. J. Med. 2018; 379:1599–1611. 30334693 10.1056/NEJMoa1807119 PMC6277022

[2] Keating D. , Marigowda G., Burr L., Daines C., Mall M.A., McKone E.F., Ramsey B.W., Rowe S.M., Sass L.A., Tullis E. et al. VX-445-tezacaftor-ivacaftor in patients with cystic fibrosis and one or two Phe508del alleles. N. Engl. J. Med. 2018; 379:1612–1620. 30334692 10.1056/NEJMoa1807120 PMC6289290

[3] Ramsey B.W. , Davies J., McElvaney N.G., Tullis E., Bell S.C., Drevinek P., Griese M., McKone E.F., Wainwright C.E., Konstan M.W. et al. A CFTR potentiator in patients with cystic fibrosis and the G551D mutation. N. Engl. J. Med. 2011; 365:1663–1672. 22047557 10.1056/NEJMoa1105185 PMC3230303

[4] Manfredi C. , Tindall J.M., Hong J.S., Sorscher E.J. Making precision medicine personal for cystic fibrosis. Science. 2019; 365:220–221. 31320522 10.1126/science.aaw0553 PMC7060931

[5] Aslam A.A. , Higgins C., Sinha I.P., Southern K.W. Ataluren and similar compounds (specific therapies for premature termination codon class I mutations) for cystic fibrosis. Cochrane Database Syst. Rev. 2017; 1:CD012040. 28102546 10.1002/14651858.CD012040.pub2 PMC6464785

[6] Kerem E. , Hirawat S., Armoni S., Yaakov Y., Shoseyov D., Cohen M., Nissim-Rafinia M., Blau H., Rivlin J., Aviram M. et al. Effectiveness of PTC124 treatment of cystic fibrosis caused by nonsense mutations: a prospective phase II trial. Lancet. 2008; 372:719–727. 18722008 10.1016/S0140-6736(08)61168-X

[7] Aslam A. , Jahnke N., Remmington T., Southern K.W. Ataluren and similar compounds (specific therapies for premature termination codon class I mutations) for cystic fibrosis. Paediatr. Respir. Rev. 2017; 24:32–34. 28566196 10.1016/j.prrv.2017.04.001

[8] Zomer-van Ommen D.D. , Vijftigschild L.A., Kruisselbrink E., Vonk A.M., Dekkers J.F., Janssens H.M., de Winter-de Groot K.M., van der Ent C.K., Beekman J.M. Limited premature termination codon suppression by read-through agents in cystic fibrosis intestinal organoids. J. Cyst. Fibros. 2016; 15:158–162. 26255232 10.1016/j.jcf.2015.07.007

[9] Aiuti A. , Biasco L., Scaramuzza S., Ferrua F., Cicalese M.P., Baricordi C., Dionisio F., Calabria A., Giannelli S., Castiello M.C. et al. Lentiviral hematopoietic stem cell gene therapy in patients with Wiskott-Aldrich Syndrome. Science. 2013; 341:1233151. 23845947 10.1126/science.1233151 PMC4375961

[10] Nathwani A.C. , Tuddenham E.G., Rangarajan S., Rosales C., McIntosh J., Linch D.C., Chowdary P., Riddell A., Pie A.J., Harrington C. et al. Adenovirus-associated virus vector-mediated gene transfer in hemophilia B. N. Engl. J. Med. 2011; 365:2357–2365. 22149959 10.1056/NEJMoa1108046 PMC3265081

[11] Maguire A.M. , Simonelli F., Pierce E.A., Pugh E.N. Jr, Mingozzi F., Bennicelli J., Banfi S., Marshall K.A., Testa F., Surace E.M et al. Safety and efficacy of gene transfer for Leber's congenital amaurosis. N. Engl. J. Med. 2008; 358:2240–2248. 18441370 10.1056/NEJMoa0802315 PMC2829748

[12] Bennett J. , Ashtari M., Wellman J., Marshall K.A., Cyckowski L.L., Chung D.C., McCague S., Pierce E.A., Chen Y., Bennicelli J.L. et al. AAV2 gene therapy readministration in three adults with congenital blindness. Sci. Transl. Med. 2012; 4:120ra115. 10.1126/scitranslmed.3002865 PMC4169122 22323828

[13] Dunbar C.E. , High K.A., Joung J.K., Kohn D.B., Ozawa K., Sadelain M. Gene therapy comes of age. Science. 2018; 359:eaan4672. 29326244 10.1126/science.aan4672

[14] Mendell J.R. , Al-Zaidy S., Shell R., Arnold W.D., Rodino-Klapac L.R., Prior T.W., Lowes L., Alfano L., Berry K., Church K. et al. Single-dose gene-replacement therapy for spinal muscular atrophy. N. Engl. J. Med. 2017; 377:1713–1722. 29091557 10.1056/NEJMoa1706198

[15] George L.A. , Sullivan S.K., Giermasz A., Rasko J.E.J., Samelson-Jones B.J., Ducore J., Cuker A., Sullivan L.M., Majumdar S., Teitel J. et al. Hemophilia B gene therapy with a high-specific-activity factor IX variant. N. Engl. J. Med. 2017; 377:2215–2227. 29211678 10.1056/NEJMoa1708538 PMC6029626

[16] Eichler F. , Duncan C., Musolino P.L., Orchard P.J., De Oliveira S., Thrasher A.J., Armant M., Dansereau C., Lund T.C., Miller W.P. et al. Hematopoietic stem-cell gene therapy for cerebral adrenoleukodystrophy. N. Engl. J. Med. 2017; 377:1630–1638. 28976817 10.1056/NEJMoa1700554 PMC5708849

[17] Cooney A.L. , McCray P.B. Jr, Sinn P.L Cystic fibrosis gene therapy: looking back, looking forward. Genes (Basel). 2018; 9:538. 10.3390/genes9110538 PMC6266271 30405068

[18] Yan Z. , McCray P.B. Jr, Engelhardt J.F Advances in gene therapy for cystic fibrosis lung disease. Hum. Mol. Genet. 2019; 28:R88–R94. 31332440 10.1093/hmg/ddz139 PMC6796993

[19] Marquez Loza L.I. , Yuen E.C., McCray P.B. Jr Lentiviral vectors for the treatment and prevention of cystic fibrosis lung disease. Genes (Basel). 2019; 10:218. 10.3390/genes10030218 PMC6471883 30875857

[20] Alton E.W. , Beekman J.M., Boyd A.C., Brand J., Carlon M.S., Connolly M.M., Chan M., Conlon S., Davidson H.E., Davies J.C. et al. Preparation for a first-in-man lentivirus trial in patients with cystic fibrosis. Thorax. 2017; 72:137–147. 27852956 10.1136/thoraxjnl-2016-208406 PMC5284333

[21] Alton E.W. , Armstrong D.K., Ashby D., Bayfield K.J., Bilton D., Bloomfield E.V., Boyd A.C., Brand J., Buchan R., Calcedo R. et al. Repeated nebulisation of non-viral CFTR gene therapy in patients with cystic fibrosis: a randomised, double-blind, placebo-controlled, phase 2b trial. Lancet Respir. Med. 2015; 3:684–691. 26149841 10.1016/S2213-2600(15)00245-3 PMC4673100

[22] Miah K.M. , Hyde S.C., Gill D.R. Emerging gene therapies for cystic fibrosis. Expert Rev. Respir. Med. 2019; 13:709–725. 31215818 10.1080/17476348.2019.1634547

[23] Robinson E. , MacDonald K.D., Slaughter K., McKinney M., Patel S., Sun C., Sahay G. Lipid nanoparticle-delivered chemically modified mRNA restores chloride secretion in cystic fibrosis. Mol. Ther. 2018; 26:2034–2046. 29910178 10.1016/j.ymthe.2018.05.014 PMC6094356

[24] Haque A. , Dewerth A., Antony J.S., Riethmuller J., Schweizer G.R., Weinmann P., Latifi N., Yasar H., Pedemonte N., Sondo E. et al. Chemically modified hCFTR mRNAs recuperate lung function in a mouse model of cystic fibrosis. Sci. Rep. 2018; 8:16776. 30425265 10.1038/s41598-018-34960-0 PMC6233194

[25] McNeer N.A. , Anandalingam K., Fields R.J., Caputo C., Kopic S., Gupta A., Quijano E., Polikoff L., Kong Y., Bahal R. et al. Nanoparticles that deliver triplex-forming peptide nucleic acid molecules correct F508del CFTR in airway epithelium. Nat. Commun. 2015; 6:6952. 25914116 10.1038/ncomms7952 PMC4480796

[26] Jinek M. , Chylinski K., Fonfara I., Hauer M., Doudna J.A., Charpentier E. A programmable dual-RNA-guided DNA endonuclease in adaptive bacterial immunity. Science. 2012; 337:816–821. 22745249 10.1126/science.1225829 PMC6286148

[27] Mali P. , Yang L., Esvelt K.M., Aach J., Guell M., DiCarlo J.E., Norville J.E., Church G.M. RNA-guided human genome engineering via Cas9. Science. 2013; 339:823–826. 23287722 10.1126/science.1232033 PMC3712628

[28] Cong L. , Ran F.A., Cox D., Lin S., Barretto R., Habib N., Hsu P.D., Wu X., Jiang W., Marraffini L.A. et al. Multiplex genome engineering using CRISPR/Cas systems. Science. 2013; 339:819–823. 23287718 10.1126/science.1231143 PMC3795411

[29] Crane A.M. , Kramer P., Bui J.H., Chung W.J., Li X.S., Gonzalez-Garay M.L., Hawkins F., Liao W., Mora D., Choi S. et al. Targeted correction and restored function of the CFTR gene in cystic fibrosis induced pluripotent stem cells. Stem Cell Reports. 2015; 4:569–577. 25772471 10.1016/j.stemcr.2015.02.005 PMC4400651

[30] Hollywood J.A. , Lee C.M., Scallan M.F., Harrison P.T. Analysis of gene repair tracts from Cas9/gRNA double-stranded breaks in the human CFTR gene. Sci. Rep. 2016; 6:32230. 27557525 10.1038/srep32230 PMC4997560

[31] Schwank G. , Koo B.K., Sasselli V., Dekkers J.F., Heo I., Demircan T., Sasaki N., Boymans S., Cuppen E., van der Ent C.K. et al. Functional repair of CFTR by CRISPR/Cas9 in intestinal stem cell organoids of cystic fibrosis patients. Cell Stem Cell. 2013; 13:653–658. 24315439 10.1016/j.stem.2013.11.002

[32] Firth A.L. , Menon T., Parker G.S., Qualls S.J., Lewis B.M., Ke E., Dargitz C.T., Wright R., Khanna A., Gage F.H. et al. Functional gene correction for cystic fibrosis in lung epithelial cells generated from patient iPSCs. Cell. Rep. 2015; 12:1385–1390. 26299960 10.1016/j.celrep.2015.07.062 PMC4559351

[33] Valley H.C. , Bukis K.M., Bell A., Cheng Y., Wong E., Jordan N.J., Allaire N.E., Sivachenko A., Liang F., Bihler H. et al. Isogenic cell models of cystic fibrosis-causing variants in natively expressing pulmonary epithelial cells. J. Cyst. Fibros. 2019; 18:476–483. 30563749 10.1016/j.jcf.2018.12.001

[34] Vaidyanathan S. , Salahudeen A.A., Sellers Z.M., Bravo D.T., Choi S.S., Batish A., Le W., Baik R., de la O.S., Kaushik M.P. et al. High-efficiency, selection-free gene repair in airway stem cells from cystic fibrosis patients rescues CFTR function in differentiated epithelia. Cell Stem Cell. 2020; 26:161–171. 31839569 10.1016/j.stem.2019.11.002 PMC10908575

[35] Maule G. , Casini A., Montagna C., Ramalho A.S., De Boeck K., Debyser Z., Carlon M.S., Petris G., Cereseto A. Allele specific repair of splicing mutations in cystic fibrosis through AsCas12a genome editing. Nat. Commun. 2019; 10:3556. 31391465 10.1038/s41467-019-11454-9 PMC6685978

[36] Sanz D.J. , Hollywood J.A., Scallan M.F., Harrison P.T. Cas9/gRNA targeted excision of cystic fibrosis-causing deep-intronic splicing mutations restores normal splicing of CFTR mRNA. PLoS One. 2017; 12:e0184009. 28863137 10.1371/journal.pone.0184009 PMC5581164

[37] Alapati D. , Morrisey E.E. Gene editing and genetic lung disease. Basic research meets therapeutic application. Am. J. Respir. Cell Mol. Biol. 2017; 56:283–290. 27780343 10.1165/rcmb.2016-0301PS PMC5359541

[38] Lee C.M. , Flynn R., Hollywood J.A., Scallan M.F., Harrison P.T. Correction of the DeltaF508 mutation in the cystic fibrosis transmembrane conductance regulator gene by zinc-finger nuclease homology-directed repair. Biores. Open Access. 2012; 1:99–108. 23514673 10.1089/biores.2012.0218 PMC3559198

[39] Suzuki S. , Crane A.M., Anirudhan V., Barilla C., Matthias N., Randell S.H., Rab A., Sorscher E.J., Kerschner J.L., Yin S. et al. Highly efficient gene editing of cystic fibrosis patient-derived airway basal cells results in functional CFTR correction. Mol. Ther. 2020; 28:1684–1695. 32402246 10.1016/j.ymthe.2020.04.021 PMC7335734

[40] Lin S. , Staahl B.T., Alla R.K., Doudna J.A. Enhanced homology-directed human genome engineering by controlled timing of CRISPR/Cas9 delivery. eLife. 2014; 3:e04766. 25497837 10.7554/eLife.04766 PMC4383097

[41] Wang G. , Slepushkin V.A., Zabner J., Keshavjee S., Johnston J.C., Sauter S.L., Jolly D.J., Dubensky T., Davidson B.L., McCray P.B. Jr Feline immunodeficiency virus vectors persistently transduce nondividing airway epithelia and correct the cystic fibrosis defect. J. Clin. Invest. 1999; 104:R49–R56. 10587528 10.1172/JCI8390 PMC483477

[42] Leigh M.W. , Kylander J.E., Yankaskas J.R., Boucher R.C. Cell proliferation in bronchial epithelium and submucosal glands of cystic fibrosis patients. Am. J. Respir. Cell. Mol. Biol. 1995; 12:605–612. 7766425 10.1165/ajrcmb.12.6.7766425

[43] Yeh W.H. , Chiang H., Rees H.A., Edge A.S.B., Liu D.R. In vivo base editing of post-mitotic sensory cells. Nat. Commun. 2018; 9:2184. 29872041 10.1038/s41467-018-04580-3 PMC5988727

[44] Komor A.C. , Kim Y.B., Packer M.S., Zuris J.A., Liu D.R. Programmable editing of a target base in genomic DNA without double-stranded DNA cleavage. Nature. 2016; 533:420–424. 27096365 10.1038/nature17946 PMC4873371

[45] Gaudelli N.M. , Komor A.C., Rees H.A., Packer M.S., Badran A.H., Bryson D.I., Liu D.R. Programmable base editing of A*T to G*C in genomic DNA without DNA cleavage. Nature. 2017; 551:464–471. 29160308 10.1038/nature24644 PMC5726555

[46] Mok B.Y. , de Moraes M.H., Zeng J., Bosch D.E., Kotrys A.V., Raguram A., Hsu F., Radey M.C., Peterson S.B., Mootha V.K. et al. A bacterial cytidine deaminase toxin enables CRISPR-free mitochondrial base editing. Nature. 2020; 583:631–637. 32641830 10.1038/s41586-020-2477-4 PMC7381381

[47] Anzalone A.V. , Koblan L.W., Liu D.R. Genome editing with CRISPR-Cas nucleases, base editors, transposases and prime editors. Nat. Biotechnol. 2020; 38:824–844. 32572269 10.1038/s41587-020-0561-9

[48] Liang P. , Ding C., Sun H., Xie X., Xu Y., Zhang X., Sun Y., Xiong Y., Ma W., Liu Y. et al. Correction of beta-thalassemia mutant by base editor in human embryos. Protein & Cell. 2017; 8:811–822. 28942539 10.1007/s13238-017-0475-6 PMC5676594

[49] Zeng Y. , Li J., Li G., Huang S., Yu W., Zhang Y., Chen D., Chen J., Liu J., Huang X. Correction of the Marfan syndrome pathogenic FBN1 mutation by base editing in human cells and heterozygous embryos. Mol. Ther. 2018; 26:2631–2637. 30166242 10.1016/j.ymthe.2018.08.007 PMC6224777

[50] Miller S.M. , Wang T., Randolph P.B., Arbab M., Shen M.W., Huang T.P., Matuszek Z., Newby G.A., Rees H.A., Liu D.R. Continuous evolution of SpCas9 variants compatible with non-G PAMs. Nat. Biotechnol. 2020; 38:471–481. 32042170 10.1038/s41587-020-0412-8 PMC7145744

[51] Walton R.T. , Christie K.A., Whittaker M.N., Kleinstiver B.P. Unconstrained genome targeting with near-PAMless engineered CRISPR-Cas9 variants. Science. 2020; 368:290–296. 32217751 10.1126/science.aba8853 PMC7297043

[52] Kim Y.B. , Komor A.C., Levy J.M., Packer M.S., Zhao K.T., Liu D.R. Increasing the genome-targeting scope and precision of base editing with engineered Cas9-cytidine deaminase fusions. Nat. Biotechnol. 2017; 35:371–376. 28191901 10.1038/nbt.3803 PMC5388574

[53] Richter M.F. , Zhao K.T., Eton E., Lapinaite A., Newby G.A., Thuronyi B.W., Wilson C., Koblan L.W., Zeng J., Bauer D.E. et al. Phage-assisted evolution of an adenine base editor with improved Cas domain compatibility and activity. Nat. Biotechnol. 2020; 38:883–891. 32433547 10.1038/s41587-020-0453-z PMC7357821

[54] Thuronyi B.W. , Koblan L.W., Levy J.M., Yeh W.H., Zheng C., Newby G.A., Wilson C., Bhaumik M., Shubina-Oleinik O., Holt J.R. et al. Continuous evolution of base editors with expanded target compatibility and improved activity. Nat. Biotechnol. 2019; 37:1070–1079. 31332326 10.1038/s41587-019-0193-0 PMC6728210

[55] Tsai S.Q. , Zheng Z., Nguyen N.T., Liebers M., Topkar V.V., Thapar V., Wyvekens N., Khayter C., Iafrate A.J., Le L.P. et al. GUIDE-seq enables genome-wide profiling of off-target cleavage by CRISPR-Cas nucleases. Nat. Biotechnol. 2015; 33:187–197. 25513782 10.1038/nbt.3117 PMC4320685

[56] Jin S. , Zong Y., Gao Q., Zhu Z., Wang Y., Qin P., Liang C., Wang D., Qiu J.L., Zhang F. et al. Cytosine, but not adenine, base editors induce genome-wide off-target mutations in rice. Science. 2019; 364:292–295. 30819931 10.1126/science.aaw7166

[57] Zuo E. , Sun Y., Wei W., Yuan T., Ying W., Sun H., Yuan L., Steinmetz L.M., Li Y., Yang H. Cytosine base editor generates substantial off-target single-nucleotide variants in mouse embryos. Science. 2019; 364:289–292. 30819928 10.1126/science.aav9973 PMC7301308

[58] Doman J.L. , Raguram A., Newby G.A., Liu D.R. Evaluation and minimization of Cas9-independent off-target DNA editing by cytosine base editors. Nat. Biotechnol. 2020; 38:620–628. 32042165 10.1038/s41587-020-0414-6 PMC7335424

[59] Landrum M.J. , Lee J.M., Benson M., Brown G., Chao C., Chitipiralla S., Gu B., Hart J., Hoffman D., Hoover J. et al. ClinVar: public archive of interpretations of clinically relevant variants. Nucleic Acids Res. 2016; 44:D862–D868. 26582918 10.1093/nar/gkv1222 PMC4702865

[60] Geurts M.H. , de Poel E., Amatngalim G.D., Oka R., Meijers F.M., Kruisselbrink E., van Mourik P., Berkers G., de Winter-de Groot K.M., Michel S. et al. CRISPR-based adenine editors correct nonsense mutations in a cystic fibrosis organoid biobank. Cell Stem Cell. 2020; 26:503–510. 32084388 10.1016/j.stem.2020.01.019

[61] Jiang T. , Henderson J.M., Coote K., Cheng Y., Valley H.C., Zhang X.O., Wang Q., Rhym L.H., Cao Y., Newby G.A. et al. Chemical modifications of adenine base editor mRNA and guide RNA expand its application scope. Nat. Commun. 2020; 11:1979. 32332735 10.1038/s41467-020-15892-8 PMC7181807

[62] Huang T.P. , Zhao K.T., Miller S.M., Gaudelli N.M., Oakes B.L., Fellmann C., Savage D.F., Liu D.R. Circularly permuted and PAM-modified Cas9 variants broaden the targeting scope of base editors. Nat. Biotechnol. 2019; 37:626–631. 31110355 10.1038/s41587-019-0134-y PMC6551276

[63] Koblan L.W. , Doman J.L., Wilson C., Levy J.M., Tay T., Newby G.A., Maianti J.P., Raguram A., Liu D.R. Improving cytidine and adenine base editors by expression optimization and ancestral reconstruction. Nat. Biotechnol. 2018; 36:843–846. 29813047 10.1038/nbt.4172 PMC6126947

[64] Nishimasu H. , Shi X., Ishiguro S., Gao L., Hirano S., Okazaki S., Noda T., Abudayyeh O.O., Gootenberg J.S., Mori H. et al. Engineered CRISPR-Cas9 nuclease with expanded targeting space. Science. 2018; 361:1259–1262. 30166441 10.1126/science.aas9129 PMC6368452

[65] Zabner J. , Karp P., Seiler M., Phillips S.L., Mitchell C.J., Saavedra M., Welsh M., Klingelhutz A.J. Development of cystic fibrosis and non-cystic fibrosis airway cell lines. Am. J. Physiol. 2003; 284:L844–L854. 10.1152/ajplung.00355.2002 12676769

[66] Karp P.H. , Moninger T.O., Weber S.P., Nesselhauf T.S., Launspach J.L., Zabner J., Welsh M.J. Wise C. Epithelial Cell Culture Protocols. 2002; 188:Humana Press 115–137. 10.1385/1-59259-185-X:115 11987537

[67] Clement K. , Rees H., Canver M.C., Gehrke J.M., Farouni R., Hsu J.Y., Cole M.A., Liu D.R., Joung J.K., Bauer D.E. et al. CRISPResso2 provides accurate and rapid genome editing sequence analysis. Nat. Biotechnol. 2019; 37:224–226. 30809026 10.1038/s41587-019-0032-3 PMC6533916

[68] Haeussler M. , Schonig K., Eckert H., Eschstruth A., Mianne J., Renaud J.B., Schneider-Maunoury S., Shkumatava A., Teboul L., Kent J. et al. Evaluation of off-target and on-target scoring algorithms and integration into the guide RNA selection tool CRISPOR. Genome Biol. 2016; 17:148. 27380939 10.1186/s13059-016-1012-2 PMC4934014

[69] Cooney A.L. , Abou Alaiwa M.H., Shah V.S., Bouzek D.C., Stroik M.R., Powers L.S., Gansemer N.D., Meyerholz D.K., Welsh M.J., Stoltz D.A. et al. Lentiviral- mediated phenotypic correction of cystic fibrosis pigs. JCI Insight. 2016; 1:e88730. 10.1172/jci.insight.88730 PMC5027966 27656681

[70] Dork T. , Macek M. Jr, Mekus F., Tummler B., Tzountzouris J., Casals T., Krebsova A., Koudova M., Sakmaryova I., Macek M. Sr et al. Characterization of a novel 21-kb deletion, CFTRdele2,3(21 kb), in the CFTR gene: a cystic fibrosis mutation of Slavic origin common in Central and East Europe. Hum. Genet. 2000; 106:259–268. 10798353 10.1007/s004390000246

[71] Levy J.M. , Yeh W.H., Pendse N., Davis J.R., Hennessey E., Butcher R., Koblan L.W., Comander J., Liu Q., Liu D.R. Cytosine and adenine base editing of the brain, liver, retina, heart and skeletal muscle of mice via adeno-associated viruses. Nat. Biomed. Eng. 2020; 4:97–110. 31937940 10.1038/s41551-019-0501-5 PMC6980783

[72] Cutting G.R. Cystic fibrosis genetics: from molecular understanding to clinical application. Nat. Rev.. Genet. 2015; 16:45–56. 25404111 10.1038/nrg3849 PMC4364438

[73] Highsmith W.E. , Burch L.H., Zhou Z., Olsen J.C., Boat T.E., Spock A., Gorvoy J.D., Quittel L., Friedman K.J., Silverman L.M. et al. A novel mutation in the cystic fibrosis gene in patients with pulmonary disease but normal sweat chloride concentrations. N. Engl. J. Med. 1994; 331:974–980. 7521937 10.1056/NEJM199410133311503

[74] Stewart B. , Zabner J., Shuber A.P., Welsh M.J., McCray P.B. Jr Normal sweat chloride values do not exclude the diagnosis of cystic fibrosis. Am. J. Respir. Crit. Care Med. 1995; 151:899–903. 7533604 10.1164/ajrccm/151.3_Pt_1.899

[75] Shah V.S. , Ernst S., Tang X.X., Karp P.H., Parker C.P., Ostedgaard L.S., Welsh M.J. Relationships among CFTR expression, HCO3- secretion, and host defense may inform gene- and cell-based cystic fibrosis therapies. PNAS. 2016; 113:5382–5387. 27114540 10.1073/pnas.1604905113 PMC4868420

[76] Montoro D.T. , Haber A.L., Biton M., Vinarsky V., Lin B., Birket S.E., Yuan F., Chen S., Leung H.M., Villoria J. et al. A revised airway epithelial hierarchy includes CFTR-expressing ionocytes. Nature. 2018; 560:319–324. 30069044 10.1038/s41586-018-0393-7 PMC6295155

[77] Plasschaert L.W. , Zilionis R., Choo-Wing R., Savova V., Knehr J., Roma G., Klein A.M., Jaffe A.B. A single-cell atlas of the airway epithelium reveals the CFTR-rich pulmonary ionocyte. Nature. 2018; 560:377–381. 30069046 10.1038/s41586-018-0394-6 PMC6108322

[78] Okuda K. , Dang H., Kobayashi Y., Carraro G., Nakano S., Chen G., Kato T., Asakura T., Gilmore R.C., Morton L.C. et al. Secretory cells dominate airway CFTR expression and function in human airway superficial epithelia. Am. J. Respir. Crit. Care Med. 2020; 203:1275–1289. 10.1164/rccm.202008-3198OC PMC8456462 33321047

[79] Gehrke J.M. , Cervantes O., Clement M.K., Wu Y., Zeng J., Bauer D.E., Pinello L., Joung J.K. An APOBEC3A-Cas9 base editor with minimized bystander and off-target activities. Nat. Biotechnol. 2018; 36:977–982. 30059493 10.1038/nbt.4199 PMC6181770

[80] Krishnamurthy S. , Wohlford-Lenane C., Kandimalla S., Sartre G., Meyerholz D.K., Theberge V., Hallee S., Duperre A.M., Del’Guidice T., Lepetit-Stoffaes J.P. et al. Engineered amphiphilic peptides enable delivery of proteins and CRISPR-associated nucleases to airway epithelia. Nat. Commun. 2019; 10:4906. 31659165 10.1038/s41467-019-12922-y PMC6817825

[81] Del’Guidice T. , Lepetit-Stoffaes J.P., Bordeleau L.J., Roberge J., Theberge V., Lauvaux C., Barbeau X., Trottier J., Dave V., Roy D.C. et al. Membrane permeabilizing amphiphilic peptide delivers recombinant transcription factor and CRISPR-Cas9/Cpf1 ribonucleoproteins in hard-to-modify cells. PLoS One. 2018; 13:e0195558. 29617431 10.1371/journal.pone.0195558 PMC5884575

[82] Mutyam V. , Du M., Xue X., Keeling K.M., White E.L., Bostwick J.R., Rasmussen L., Liu B., Mazur M., Hong J.S. et al. Discovery of clinically approved agents that promote suppression of cystic fibrosis transmembrane conductance regulator nonsense mutations. Am. J. Respir. Crit. Care Med. 2016; 194:1092–1103. 27104944 10.1164/rccm.201601-0154OC PMC5114449

[83] Crawford D.K. , Mullenders J., Pott J., Boj S.F., Landskroner-Eiger S., Goddeeris M.M. Targeting G542X CFTR nonsense alleles with ELX-02 restores CFTR function in human-derived intestinal organoids. J. Cyst. Fibros. 2021; 20:436–442. 33558100 10.1016/j.jcf.2021.01.009

[84] Michaels W.E. , Bridges R.J., Hastings M.L. Antisense oligonucleotide-mediated correction of CFTR splicing improves chloride secretion in cystic fibrosis patient-derived bronchial epithelial cells. Nucleic Acids Res. 2020; 48:7454–7467. 32520327 10.1093/nar/gkaa490 PMC7367209

[85] Chen H. , Matsumoto K., Brockway B.L., Rackley C.R., Liang J., Lee J.H., Jiang D., Noble P.W., Randell S.H., Kim C.F. et al. Airway epithelial progenitors are region specific and show differential responses to bleomycin-induced lung injury. Stem Cells. 2012; 30:1948–1960. 22696116 10.1002/stem.1150 PMC4083019

[86] Rock J.R. , Onaitis M.W., Rawlins E.L., Lu Y., Clark C.P., Xue Y., Randell S.H., Hogan B.L. Basal cells as stem cells of the mouse trachea and human airway epithelium. Proc. Natl. Acad. Sci. U.S.A. 2009; 106:12771–12775. 19625615 10.1073/pnas.0906850106 PMC2714281

[87] Staudt M.R. , Buro-Auriemma L.J., Walters M.S., Salit J., Vincent T., Shaykhiev R., Mezey J.G., Tilley A.E., Kaner R.J., Ho M.W. et al. Airway Basal stem/progenitor cells have diminished capacity to regenerate airway epithelium in chronic obstructive pulmonary disease. Am. J. Respir. Crit. Care Med. 2014; 190:955–958. 25317467 10.1164/rccm.201406-1167LE PMC4299582

[88] Chapman H.A. , Li X., Alexander J.P., Brumwell A., Lorizio W., Tan K., Sonnenberg A., Wei Y., Vu T.H. Integrin alpha6beta4 identifies an adult distal lung epithelial population with regenerative potential in mice. J. Clin. Invest. 2011; 121:2855–2862. 21701069 10.1172/JCI57673 PMC3223845

[89] Li X. , Rossen N., Sinn P.L., Hornick A.L., Steines B.R., Karp P.H., Ernst S.E., Adam R.J., Moninger T.O., Levasseur D.N. et al. Integrin alpha6beta4 identifies human distal lung epithelial progenitor cells with potential as a cell-based therapy for cystic fibrosis lung disease. PLoS One. 2013; 8:e83624. 24349537 10.1371/journal.pone.0083624 PMC3861522

[90] Rawlins E.L. , Hogan B.L. Ciliated epithelial cell lifespan in the mouse trachea and lung. Am. J. Physiol. 2008; 295:L231–L234. 10.1152/ajplung.90209.2008 PMC2494792 18487354

