# Supplementary material for: Functional correction of CFTR mutations in human airway epithelial cells using adenine base editors
Source: Nucleic Acids Res. 2021 Sep 14;49(18):10558–72. doi: 10.1093/nar/gkab788 (PMC8501978; doi:10.1093/nar/gkab788)
Supplement: gkab788_Supplemental_File [file gkab788_supplemental_file.pdf]

**Supplementary Material**

# Functional Correction of *CFTR* Mutations in Human Airway Epithelial Cells using Adenine Base Editors

Sateesh Krishnamurthy[1], Soumba Traore[1], Ashley L. Cooney[1], Christian M. Brommel[1,2], Katarina Kulhankova[1], Patrick L. Sinn[1,2], Gregory A. Newby[3,4,5], David R. Liu[3,4,5], Paul B. McCray, Jr.[1,2*]

[1] Department of Pediatrics, [2] Molecular Medicine Graduate Program,
Pappajohn Biomedical Institute,
University of Iowa, Iowa City, IA, USA
[3] Merkin Institute of Transformative Technologies in Healthcare, Broad Institute of Harvard and MIT,
Cambridge, Massachusetts, USA
[4] Department of Chemistry and Chemical Biology, Harvard University, Cambridge, Massachusetts, USA
[5] Howard Hughes Medical Institute, Harvard University, Cambridge, Massachusetts, USA

*Corresponding author: Paul B. McCray, Jr., email: paul-mccray@uiowa.edu

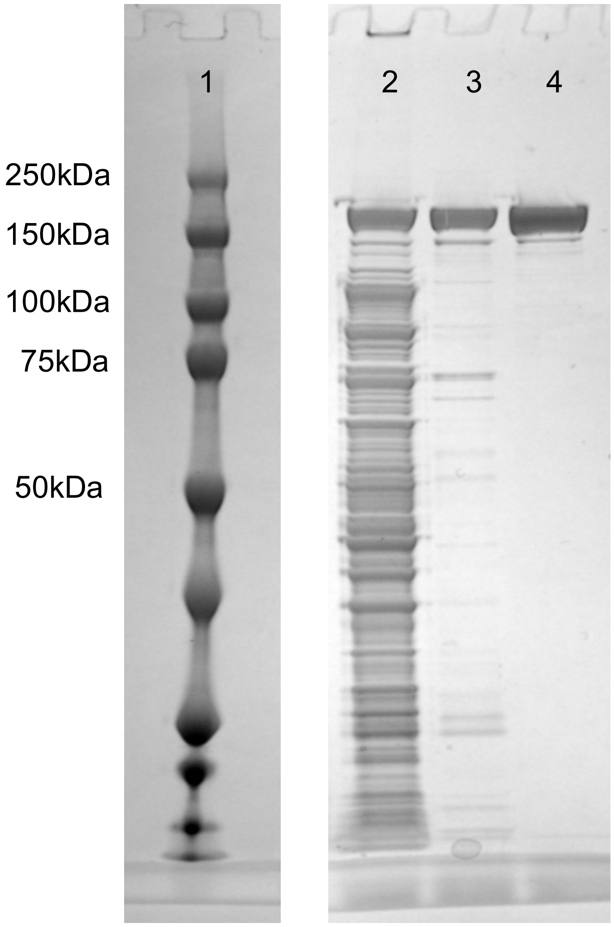


**Supplemental Figure 1** Purification of recombinant SpCas9-NG+5 nuclease. Following ion exchange chromatography, protein purity was assessed on an SDS-PAGE. Lane 1: 10 µl of Precision Plus Protein Dual Color ladder (Bio-Rad). Lane 2: 0.5 µl of lysate after nickel binding. Lane 3: 0.25 µl of nickel elution. Lane 4: 0.1 µl of concentrated protein stock after ion exchange.

A

| Target | gRNA sequence | PAM | Gene; Locus Description |
|---|---|---|---|
| R553X On Target | TTGCTCATTGACCTCCACTC | AGT | CFTR; Exon 12 |
| OT1 | **GA**G**T**TCA**C**TGACCTCCACTC | TGG | Intergenic; Y_RNA-PBX1 |
| OT2 | **G**TGCTC**G**TT**T**ACCTC**A**ACTC | TGG | Intergenic; RP11-115N4.1-RP11-142A5.1 |
| OT3 | T**G**GCTCA**C**TG**G**C**T**TCCACTC | TGG | Intron; CCDC69 |
| OT4 | TTG**AC**C**TC**TGACCTCCACTC | AGG | Intron; LLGL1 |
| OT5 | **AG**GCTC**CC**TGACCTCCACTC | TGG | Intron; CTD-2644I21.1 |
| OT6 | **C**TGCT**T**A**C**TG**C**CCTCCACTC | AGG | Intron; RP11-718O11.1 |
| OT7 | T**G**GCTC**TC**TGA**A**CTCCACTC | TGG | Intergenic; AP005118.1-PTPRM |
| OT8 | **C**TGCTCA**A**TGA**TT**TCCACTC | TGG | Exon; CCDC28B |
| OT9 | TT**T**CT**A**ATTGA**A**CTCCA**T**TC | AGG | Intron; TMEM74 |
| OT10 | TTGCTC**CC**TG**G**C**T**TCCACTC | AGG | Intergenic; EEF1GP7-GPX7 |
| Target | gRNA sequence | PAM | Gene; Locus Description |
| W1282X On Target | CAGTGAAGGAAAGCCTTTGG | AGT | CFTR; Exon 23 |
| OT1 | **AT**GTGAAGGAAAGCC**A**TT**A**G | AGG | Intron; TPK1 |
| OT2 | **A**AGTG**T**AGGAAAGCC**AC**TGG | GGG | Intergenic; RP11-278H7.3-C1orf100 |
| OT3 | CAGTG**T**AGGAAAGCC**A**TT**AA** | TGG | Intron; SCHIP1 |
| OT4 | C**G**GT**C**AAGGAAAGCC**A**T**A**GG | AGG | Exon; RP11-432J22.2 |
| OT5 | CAGTGAA**T**G**T**AAGCCT**C**TG**A** | TGG | Intergenic; AC068718.1-ST13P2 |
| OT6 | **A**AGTGAAG**C**AA**G**GCC**A**TTGG | TGG | Intergenic; TMEM178B-RP11-744I24.3 |
| OT7 | CAGTGAA**AT**AAAGCC**A**TT**A**G | TGG | Intron; FAM208A |
| OT8 | CAGT**AT**A**A**GAAAGCCT**A**TGG | AGG | Intron; SV2C |
| OT9 | **G**AGTG**TG**GGAAAGCCTTT**A**G | TGG | Exon; ZNF585B |
| OT10 | CAGTG**T**AGGAAAG**T**CTTT**TA** | GGG | Intergenic; FAM19A2-RPS3P6 |
| Target | gRNA sequence | PAM | Gene; Locus Description |
| 3849C>T+10kb On Target | GGTGAGTAAGACACCCTGAA | AGG | CFTR; Intron 22 |
| OT1 | **A**GTGA**A**TAA**AG**CACCCTGAA | AGG | Intron; MGST1 |
| OT2 | GGTGA**A**T**GT**GACACC**A**TGAA | TGG | Intergenic; INPP4B-RP11-284M14.1 |
| OT3 | **A**GTGAG**G**AA**A**ACACCCT**A**AA | AGG | Intron; EP400NL |
| OT4 | **T**GTG**G**GTA**T**GA**A**ACCCTGAA | AGG | Exon; NAV1 |
| OT5 | **AA**TGAGTAAGACA**T**CC**A**GAA | GGG | Intergenic; TMCC3-KRT19P2/MIR492 |
| OT6 | **A**G**G**GA**A**TAAG**G**CACCCTGAA | GGG | Intergenic; STXBP6-RP11-89K22.1 |
| OT7 | G**AA**GA**A**T**T**AGACACCCTGAA | CGG | Intergenic; RNU6-766P-CERS6 |
| OT8 | GGTG**G**G**C**AAGA**T**ACCCTGA**G** | GGG | Intron; GDA |
| OT9 | **A**GTGAGT**C**A**TG**CACCCTGAA | GGG | Intron; ELAVL1 |
| OT10 | GG**GA**AG**C**AAG**G**CACCCTGAA | TGG | Intergenic; RP11-555H23.1-SNORA20 |

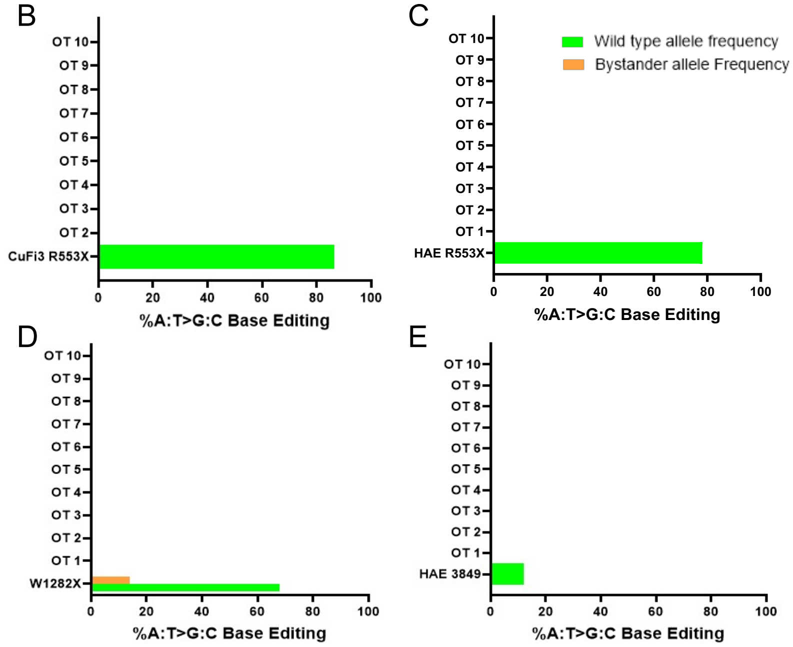


**Supplemental Figure 2** Off target analysis for adenine base editing. (A) The CRISPOR tool was used to computationally identify candidate gRNA-dependent off-target (OT) editing sites for R553X, W1282X, and 3849C>T+10kb guide RNAs and genomic OT loci. The top 10 sequences were investigated. Mismatches between on and off target sequences are denoted with bold lettering. The underlined bases are those that could be edited by ABE7.10. (B) Next generation sequencing assessed OT base editing in CuFi-3, a R553X cell line. The intergenic OT1 site in the CuFi-3 cell line did not sequence. (C,D,E) OT sites were also analyzed using next generation sequencing in R553X, W1282X, and 3849C>T+10kb primary human airway epithelia (HAE).

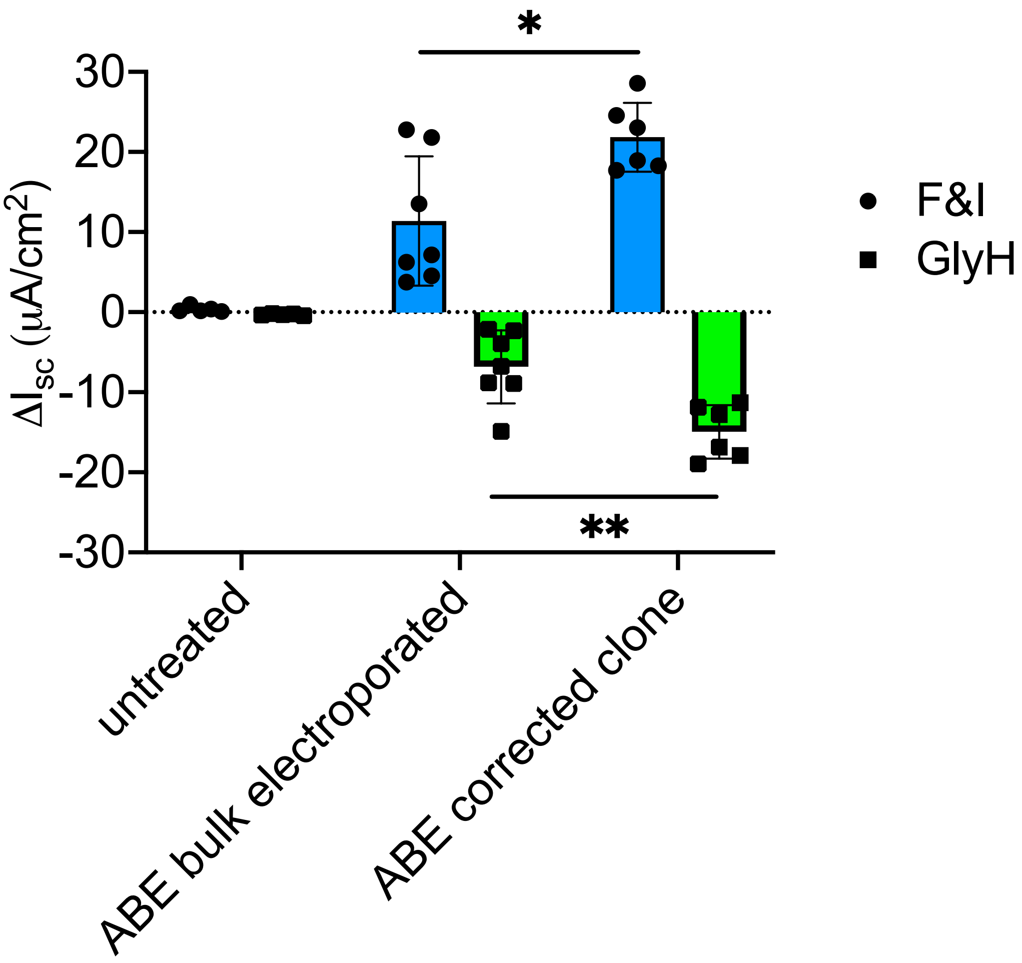


**Supplemental Figure 3** Comparison of CFTR-dependent chloride secretion in bulk edited versus a clonally selected population of ABE edited CuFi-3 cells. Following bulk ABE electroporation, CuFi-3 cells were single cell cloned and expanded to quantify $Cl^-$ secretion with bulk electroporated cells. Results are shown in replicate from epithelia derived from a clonally derived population of correctly edited cells (n=6). Compared to bulk electroporated CuFi-3 cells, the cells with a the corrected R553X allele exhibited a significantly greater CFTR-dependent $Cl^-$ current, similar to non-CF airway epithelial cells (see Figure 4). The data from bulk electroporated cells are from Figure 4. * $P < 0.04$ and ** $P < 0.004$.
